# Iron to Cobalt Swapping in a Bioinspired Heme-Peroxidase: Structural Characterization and Functional Implications

**DOI:** 10.1021/acs.inorgchem.5c05553

**Published:** 2026-04-23

**Authors:** Marco Chino, Corinne Cerrone, Fabio Pirro, Maria De Fenza, Serhiy Demeshko, Ornella Maglio, Franc Meyer, Angela Lombardi

**Affiliations:** † Department of Chemical Sciences, University of Naples Federico II, Via Cintia 21, Napoli 80126, Italy; ‡ Institute of Biostructures and Bioimaging (IBB), National Research Council (CNR), Via P. Castellino, Napoli 80131, Italy; § Institute of Inorganic Chemistry, University of Göttingen, Tammannstraße 4, Göttingen 37077, Germany

## Abstract

Artificial heme enzymes offer unique opportunities to disentangle structure–function relationships and design novel biocatalysts. Mimochromes (MCs) are artificial, small-sized heme proteins able to reproduce the structural and functional features of natural heme enzymes. Here, we report the spectroscopic and structural investigation of a Mimochrome VI (MC6) analogue, Lys9DabMC6*a, for which we were previously able to isolate two distinct regioisomers. Mössbauer and EPR spectroscopy revealed distinct pH-dependent high-spin and quantum mixed-spin states in the Fe­(III) complexes for both regioisomers. A detailed structural characterization was performed by NMR spectroscopy on the diamagnetic Co­(III) analogues, providing high-resolution structures of the two isolated regioisomers. Both species show the intended helix-heme-helix sandwich fold but differ in interhelical orientation, axial histidine positioning, and second-sphere interactions, despite having the same peptide composition. Based on the reported electronic properties and structural features, we retrospectively attempt to elucidate the differences in substrate affinity and turnover frequency between the two regioisomers. Our results provide useful insights for the rational evolution of heme-based artificial minienzymes and highlight the minimal determinants required to achieve catalytic diversity.

## Graphical abstract

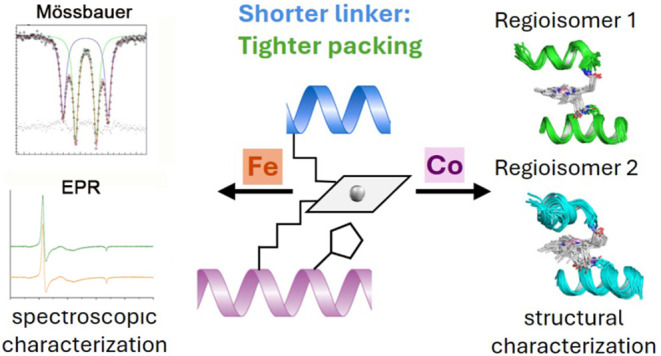

## Introduction

The construction of artificial metalloenzymes still represents a notable challenge in chemistry. Initially aimed at understanding the complex and tightly orchestrated functions of natural enzymes, nowadays it is mainly dedicated to tailoring new biocatalysts for specific applications. This goal is typically accomplished by properly designing the first- and second-coordination sphere interactions that tune the metal ion properties^1–4^ and/or induce substrate/product specificity, thereby contributing to impart biocatalyst specificity and selectivity.^5–9^ Inspired by nature, we tackled the challenge to develop metalloenzyme mimics by using peptide- and protein-based ligands,^10,11^ thus mimicking both the structural and functional features of their inspiring natural counterparts.

Among metalloenzymes, the functional diversity of heme has prompted numerous scientists to uncover the structural features that may govern a specific activity, and a variety of strategies have been used for developing functional heme-protein mimics. These strategies have provided synthetic heme-protein models, in which the metal cofactor is enclosed within a protein scaffold of different structures and functional complexities.^12–16^

Along these lines, we have developed, through a miniaturization approach, artificial small-sized heme proteins named Mimochromes (MCs). Their designed structure consists of two short helical peptides, covalently linked to deuteroporphyrin IX (DPIX) through amide bonds between the DPIX propionic groups and the ε-amino groups of Lys side chains.^11^ The two peptide chains surround the porphyrin ring in a sandwich arrangement. Over the years, we have rationally evolved the Mimochrome scaffolds, from simple redox-active metalloproteins to highly efficient peroxidases and peroxygenases.^11,17^ Starting from symmetrical *bis*-His derivatives^18,19^ and applying an iterative redesign process,^20^ we built MC6*a, an asymmetrical molecule with two different peptide chains: a proximal tetradecapeptide (TD chain) and a distal decapeptide (D chain). The TD chain contains the His axial ligand, while the D chain lacks the metal-coordinating residue, thus harboring a substrate-binding pocket on the distal side of the heme (Figure 1a).

**Figure 1:**
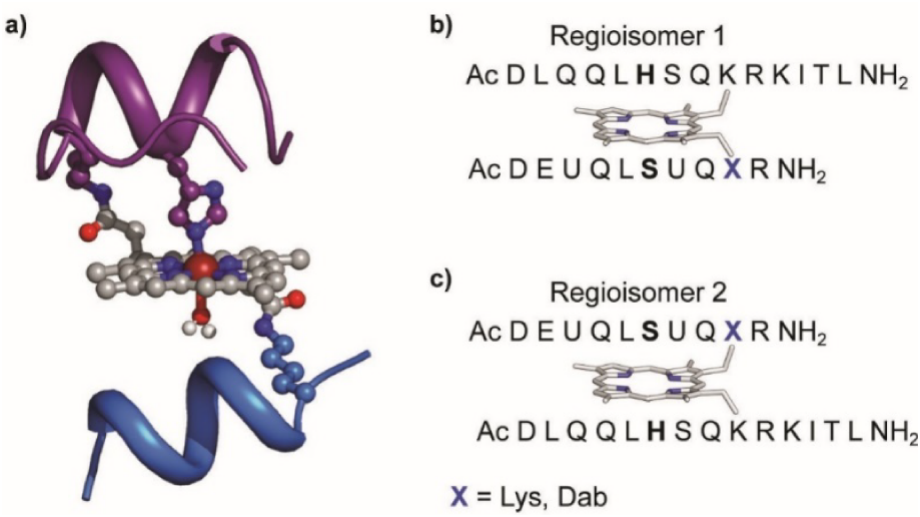
(a) MC6*a designed model showing the helix-heme-helix sandwiched motif. Backbone is represented as a ribbon, side chains of functional and structural residues are depicted as balls and sticks, and the metal ion is depicted as a red ball. D and TD chains are depicted in blue and violet, respectively. (b,c) Amino acid sequences of MC6*a regioisomers. Residues in axial positions are indicated in bold black. U indicates the Aib residues, and X indicates Lys or Dab anchoring residues.

These structural features directed Fe­(III)-MC6*a toward peroxidase catalysis and made it one of the best artificial peroxidase reported to date, exceeding the catalytic efficiency of the natural horseradish peroxidase (HRP).^20^ Noteworthy, the stable and small scaffold is proficient in modulating the reactivity of the coordinated metal ion. Indeed, MC6*a switches its activity to peroxygenase or hydrogenase when iron is replaced by manganese or cobalt, respectively.^17,21,22^

MC6*a has been thoroughly characterized in its function, but detailed structural characterization is lacking. The asymmetry of the DPIX and the different lengths and compositions of the peptide chains give rise to the simultaneous presence of two different regioisomers (Figure 1b and c). Indeed, each of the two TD and D chains can be anchored either to propionate 2 or to propionate 18 (Figure S1), giving rise to regioisomer formation. Furthermore, the flexibility of the linkers between the peptide and DP ring allows each peptide chain to be positioned either above or below the porphyrin plane, giving rise to Δ and Λ diastereomers, as previously characterized in the symmetric *bis-*His MC1 prototype.^19^ All of these phenomena lead to the coexistence of up to four species in solution (Figure 2), thus making structural insight very difficult.

**Figure 2:**
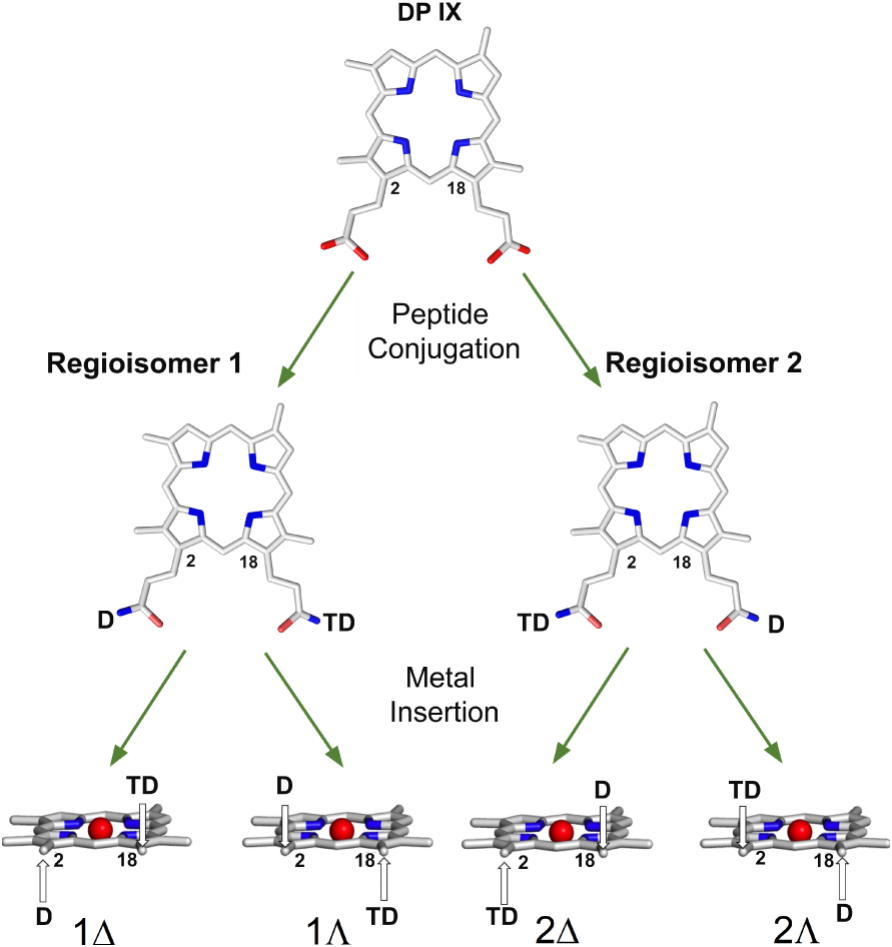
Schematic representation of Co­(III)-MC6*a diastereoisomers formation. The decapeptide chain and the tetradecapeptide chain are indicated as D and TD, respectively. The cobalt ion is shown as a red sphere.

High-resolution structural information is crucial for attaining molecular-level snapshots of synthetic metalloenzymes and can deeply contribute to understanding their reactivity. To progress with structural data, we redesigned MC6*a to strengthen the interactions between the decapeptide and the porphyrin ring.^23^ We shortened the alkyl chain that connects the distal chain to the porphyrin, obtaining an MC6*a analogue, named Lys9DabMC6*a. The lysine-to-diaminobutyric acid (Dab) substitution on the D chain enabled us to separate the two regioisomers (**1** and **2** in Figure 2) and to perform a structural analysis of the Fe­(III)-hexa-coordinated cyanide complexes by means of paramagnetic NMR shifts. We demonstrated that the two regioisomers, both adopting the Δ configuration, in the presence of trifluoroethanol (TFE), differ in the His axial-ligand orientation, which, in turn, correlates with catalytic performance.^23^

Here, we report a combined analysis of paramagnetic Fe­(III)-Lys9DabMC6*a **1Δ** and **2Δ** by EPR and Mössbauer spectroscopies, coupled with a structural characterization of the diamagnetic Co­(III) species by NMR spectroscopy. These studies allowed us to define the structural landscape for the Mimochrome class of synthetic enzymes and to pinpoint the subtle interactions required to attain high activity.

## Experimental Section

### General Materials and Methods

All solvents and TFA (trifluoroacetic acid) were supplied by Romil. All buffer solutions were prepared by using HPLC-grade water (Romil); phosphate salts (mono- and dibasic) for buffer preparation and hydrogen peroxide (H_2_O_2_) solution (30% (*v*/v)) were provided by Fluka.^57^Fe metal powder was purchased from Sigma-Aldrich (95% isotopic purity). Lys9Dab-MC6*a Fe­(III)-, ^57^Fe­(III)-, and Co­(III)-complexes were synthesized and purified as previously described.^23^

Data analysis was performed by using OriginPro 8 and Kaleidagraph softwares.

### UV–vis and CD Spectroscopies

UV–vis spectra were recorded on a Cary Varian 60 Probe UV Spectrophotometer equipped with a thermostated cell holder and a magnetic stirrer. All measurements were performed at 25 °C. Quartz cuvettes with a path length of 1.0 or 0.10 cm were used for most measurements. Wavelength scans were performed from 200 to 800 nm with a 300 nm min^–1^ scan speed. All data were blank-subtracted (see Figure S2 and S3 in Section S1 for metal coordination properties).

CD measurements were carried out on a Jasco J-815 dichrograph equipped with a thermostated cell holder (JASCO, Easton, MD, USA). CD spectra were collected from 460 to 300 nm in the Soret region at 0.2 nm intervals, with a 20 nm min^–1^ scan speed, a 1 nm bandwidth, and a 16 s response time. Spectra were averaged over 5 accumulations.

### Mössbauer Measurements

Mössbauer spectra were recorded using an alternating constant acceleration WissEL Mössbauer spectrometer consisting of an MR 360 Drive Unit, an MVT-1000 velocity transducer, and an LND 45431 proportional counter mounted on a LINOS precision bench. The Mössbauers System is equipped with a ^57^Co source in a Rh matrix with an initial activity of ∼1.85 GBq (kept at room temperature) and is operated in horizontal transmission geometry with the source, absorber, and detector in a linear arrangement. A Janis SHI 850 closed-cycle helium cryostat was used for low-temperature measurements. Data acquisition was performed by using a 512-channel analyzer. Isomer shifts are given relative to iron metal at ambient temperature. Simulation and evaluation of the experimental data were performed using the *Mfit* program (E. Bill, Max-Planck Institute for Bioinorganic Chemistry, Mülheim/Ruhr, Germany).

Mössbauer spectra were collected on ^57^Fe-Lys9Dab-MC6*a isomers in the solid state (lyophilized powder from 0.1% TFA in water) and in solution (phosphate buffer- 50 mM, pH 6.5, 50% TFE (v/v)), at T = 6 K.

### EPR Characterization

EPR analysis was carried out using a Bruker Elexsys E500 CW-EPR and a Super-X microwave bridge operating at 9.3–9.5 GHz. The spectrometer was equipped with a standard Bruker X-band ER4119-SHQE cavity and a liquid helium cryostat (Oxford Instruments) for low-temperature measurements.

The spectra were simulated using the program EasySpin.^24,25^

EPR spectra were collected on both isomers in the solid state (lyophilized from acidic conditions) and in solution (phosphate buffer: 50 mM, pH 6.5, 50% TFE (v/v)), at T = 4 K; microwave frequency, 9.41 GHz; microwave power, 0.1 mW. Spectra were also acquired at 20 and 70 K without displaying significant differences, except for lower intensity.

Compared Mössbauer and EPR parameters for Fe­(III)-Lys9Dab-MC6*a isomers are reported in Table S1.

### ^1^H NMR Analysis and Structure Calculations

NMR spectra were acquired at 298 K on a Bruker Avance 600 spectrometer equipped with a triple resonance cryoprobe. Suppression of the water signal was accomplished by excitation sculpting sequence.^26^

Samples for NMR spectroscopy were prepared by dissolving weighed amounts of Co­(III)-**1Δ** and **2Δ** in 0.600 mL of H_2_O/TFE (60/40, v/v, pH 4.6). The final concentrations of the samples were 2 × 10^–4^ M and 5 × 10^–4^ M for Co­(III)-**1Δ** and **2Δ**, respectively. Chemical shifts are relative to the sodium salt of [D_4_]­3-(trimethylsilyl)­propionic-2,2,3,3-acid.

Two-dimensional NOESY (Nuclear Overhauser Effect SpectroscopY),^27,28^ TOCSY (TOtal Correlation SpectroscopY)^29,30^ and DQF-COSY (Double Quantum Filtered COrrelation SpectroscopY)^31^ spectra were acquired. The TOCSY experiment was performed with a spin lock applied for 70 ms and was used for the identification of spin systems. All spectra were acquired by using a spectral width of 8400 Hz in both dimensions. The two-dimensional data consisted of 4K data points in the direct dimension and 512 complex data points, zero-filled to 1K data points prior to Fourier transformation. Raw data were multiplied in both dimensions by using a cosine bell function before Fourier transformation.

NMR spectra were processed using Bruker TOPSPIN software, which freely available from Bruker for academic users, and analyzed with the CARA program (cara.nmr.ch/doku.php/home). The ^1^H chemical shifts (in ppm) of Co­(III)-**1Δ** and **2Δ** are reported in Section S3.

Intensities of dipolar connectivities in the NOESY spectrum, obtained using a 200 ms mixing time, were integrated with CARA software and converted into distance upper limits by following the methodology of the program CALIBA.^32^ The number of constraints, including intraresidual, sequential, medium-range, and long-range upper distance limits, is shown in Section S3. The total number of constraints was also supplemented by a set of constraints to simulate histidine coordination.

The three-dimensional structures were preliminarily calculated with the program CYANA^33^ and then refined with AMBER^34,35^ as follows. Typical CYANA runs were performed on 400 randomly generated starting structures with 10,000 torsion angle dynamics steps. The 20 CYANA structures with the lowest target function were subjected to restrained molecular dynamics (RMD) and restrained energy minimization (REM) with the Sander module of the AMBER 10.0 package.^36^ The protonation state of the coordinating His6 was determined from the NMR spectra: the slowly exchanging Nδ1H signal (9.27 ppm for **1Δ**, 9.19 ppm for **2Δ**) confirms that His6 is coordinated through Nε with Nδ1 protonated. The RMD and REM protocols were adapted from Banci and coworkers,^37^ which were previously used on natural heme proteins. Briefly, the RMD consisted of a 48 ps simulation at 300 K (Berendsen thermostat) performed *in vacuo* with a 10 Å non-bonded cutoff. NOE-derived distance restraints were applied through a flat-well potential with a parabolic penalty within 0.5 Å outside the upper bound and linear beyond, using a force constant of 32 kcal mol^–1^ Å^–2^. Restraint weights were gradually increased from 0.1 to 1.0 over the first 1000 steps. The 20 lowest-energy structures from RMD were averaged, and the resulting structure was subjected to REM using 5000 cycles of minimization (2000 steepest descent followed by conjugate gradient) with the Hawkins-Cramer-Truhlar generalized Born implicit solvent model and full restraint weight. As the RMD and REM were performed *in vacuo* and in implicit solvent, respectively, a hydroxide molecule was manually added with PyMOL^38^ to establish system electroneutrality and to saturate the coordination environment of the cobalt ion. The DPIX (with the amidated proprionic groups), the Aib residue, the Dab and Lys residues, acetylated on the side chain, were parametrized using Antechamber;^39^ the computational data are available in a Zenodo repository (https://zenodo.org/records/17019610). AMBER atom types were preferred to GAFF ones for all the residues,^40^ when this was possible. The partial charges at the tether points between the propionic groups and the peptide side chains were manually adjusted to ensure the correct overall charge after removal of the capping amide and acetyl groups. CHARMM parameters were used for the hydroxide ion, while cobalt ion was adapted from Cheatham and Kollman.^41^ The force field parameters for all the other residues were the standard AMBER ff99SB “all atom” parameters.^42^ Finally, the cobalt ion was covalently bound to the pyrrolic nitrogen atoms of the deuteroporphyrin, the Nε of the histidine residue in the tetradecapeptide chain, and the oxygen atom of the hydroxide ion. The LEaP code to prepare the starting structure for the simulation is reported in Section S4.

The quality of the obtained structures was evaluated in terms of deviations from ideal lengths and bond angles through a Ramachandran plot obtained using the program PROCHECK-NMR^43^ and the results of the analysis are reported in Section S3. NMR-derived coordinates and distance constraints are accessible on the PDB (PDB ID: 9GSA, 9GW6). Further analyses of the NMR structures were performed with PyMOL.^38^

## Results and Discussion

### Fe­(III)-Lys9Dab-MC6*a: Spectroscopic Analysis

#### UV/vis Spectroscopy

Fe­(III) was inserted into the apo forms of the two individual 1 and 2 regioisomers, as previously reported.^23^ The coordination properties of each iron-containing regioisomer were analyzed by UV/vis spectroscopy. A pH titration was performed in the range 2.0–9.0 in a 50% H_2_O/TFE (v/v) solution (Section S1). Fe­(III)-Lys9Dab-MC6*a **1** and **2** regioisomers show similar pH dependencies, in good agreement with the previously designed MC analogue.^20^

UV–vis analysis indicated the presence of three pH-dependent species: a high spin (HS) *bis*-aquo H_2_O/H_2_O species under acidic pH, a 6-coordinate His/H_2_O species approximately in the range of pH between 4.0 and 7.0, and a hydroxo complex His/OH^–^ under alkaline pH in an HS-LS equilibrium, reasonably closer to the HS (Section S1).^44,45^

### Mössbauer and EPR Spectroscopies

To further investigate the electronic fine structure and possible differences in Fe­(III)-Lys9Dab-MC6*a **1** and **2** regioisomers, ^57^Fe Mössbauer and EPR experiments were performed under different conditions.

Mössbauer spectroscopy has been frequently used to investigate the oxidation and spin state of the metal ion in iron porphyrins and hemoproteins.^46,47^ Isomer shift (δ) and quadrupole splitting (ΔE_Q_) data, measured under zero applied magnetic field, are usually sufficient to identify both the oxidation and spin states. ^57^Fe Mössbauer spectra were recorded on solid-state samples (lyophilized from acidic conditions: 0.1% TFA in water, pH ∼ 2) and on frozen solution samples (sodium phosphate buffer: 50 mM, pH 6.5, 50% TFE (v/v)) at T = 6 K. The Mössbauer spectra of powder samples display two quadrupole doublets in an approximately 1:1 ratio (53:47 for both **1** and **2**; see Figure 3a–b and Table S1 in Section S1).

**Figure 3:**
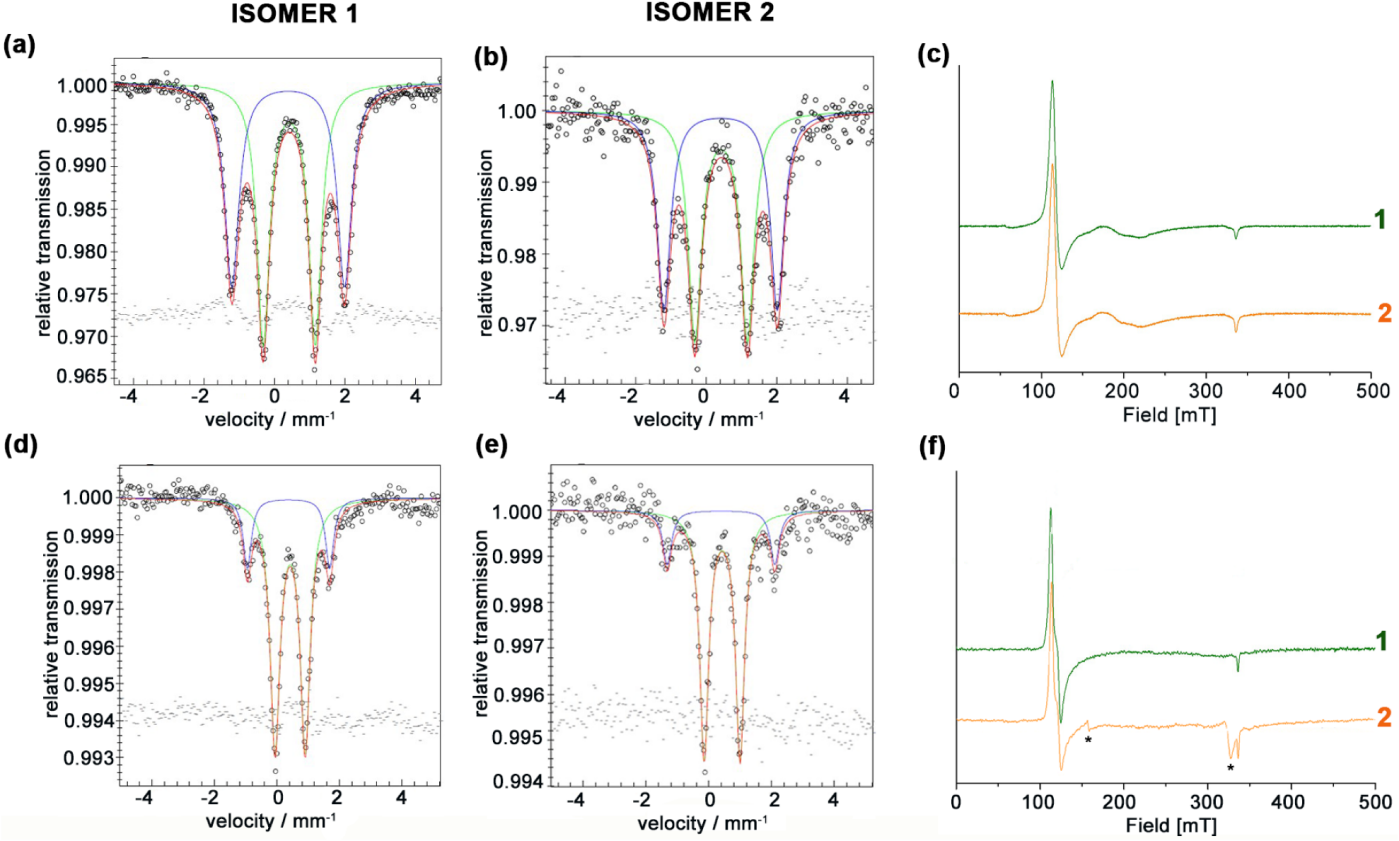
^57^Fe Mössbauer and EPR spectra of Fe­(III)-**1** and **2** regioisomers in the solid state (a, b, c) and in solution (TFE/50 mM phosphate buffer, 50% *v/v,* pH 6.5) (d, e, f). Mössbauer spectra of frozen solution samples were acquired at 3.6 × 10^–3^ and 4.7 × 10^–3^ M for **1** and **2,** respectively. Circles are the experimental data, which were fitted with a two-state model (red line, sum of green and blue lines). EPR spectra for regioisomers **1** (c, f green lines) and **2** (c, f orange lines) solution samples were acquired at 7.6 × 10^–5^ M and 9.2 × 10^–5^ M, respectively. *Cavity signal.

The first quadrupole doublet (Figure 3a–b, green line) has been observed for both regioisomers, having Mössbauer parameters [δ­(ΔE_Q_): 0.42 (1.47) mm/s and 0.43 (1.49) mm/s for **1** and **2,** respectively], indicative of six-coordinate high-spin (S = 5/2) Fe­(III)-porphyrin with weak-field ligands (see Figure S4 in Section S1), such as H_2_O.^48^ The observed quadrupole splitting values are slightly larger than those found in other high-spin six-coordinate heme derivatives, which have been correlated to deviations from octahedral symmetry due to longer axial Fe–O_wat_ bonds.^48^ The second doublet (Figure 3a–b, blue lines) shows an isomer shift very similar to that observed for the first doublet, though exhibiting a significantly higher quadrupole splitting [δ­(ΔE_Q_): = 0.40 (3.20) mm/s and 0.41 (3.21) mm/s for **1** and **2**, respectively]. These unusually high quadrupole splitting values might result from a quantum mechanical admixed-spin (QS) state (S = 3/2, 5/2), an electronic configuration that has been reported for some class III heme peroxidases of the plant peroxidase superfamily.^49^ The QS state corresponds to a single magnetic species with distinct properties from pure spin species and is not related to a thermal equilibrium between the S = 5/2 and S = 3/2 states.^50–52^ It has been noted that a conformational change in proteins, causing no substantial change in axial ligands but resulting in modifications in heme interactions and ring distortion, can induce a movement of the iron along the *z*-axis and may be responsible for the unusual spin state.^53,54^ These considerations suggest that both isomers exist as 6-coordinate Fe­(III)-porphyrin bis­(H_2_O) complexes, but in the QS and HS states. The observed HS/QS coexistence may be explained either by structural heterogeneity in the solid state (due to packing interactions in the amorphous solid phase) or as Δ*a*nd Λ diastereomeric equilibrium (favored by the protonated proximal His side chain).

Further evidence for the copresence of different spin states comes from EPR measurements. The EPR spectra of the two isomers in the solid state (Figure 3c; see Table S1 and Figure S5 in the Supporting Information) at T = 4 K are superimposable and display three signals at g ≈ 5.63, 3.60, and 2.00. EasySpin simulations of the solid-state spectra as the sum of two contributions (Figure S5 and Table S1 in Section S1) reveal a predominant (∼80%) slightly rhombic HS species with (g_*x*_, g_*y*_, g_*z*_) = (5.92, 5.58, 2.00) and E/D = 0.01, and a minor (∼20%) QS species with (g_*x*_, g_*y*_, g_*z*_) = (5.92, 3.56, 2.00) and E/D = 0.04, in agreement with the Mössbauer assignment. The discrepancy in relative intensities between Mössbauer (∼50:50) and EPR (∼80:20) is attributable to the intrinsic differences between S = 5/2 and S = 3/2 spin-transition moments. According to the Maltempo treatment,^53^ (a_5/2_)^2^ = (g_⊥_-4)/2 = 0.37, confirming a predominantly S = 3/2 character for the minor QS component (i.e., the fractional weight of the S = 5/2 component in the admixed state is ∼37%).

Even though a ferric HS state generally features g_⊥_ ≈ 6 and g_||_ ≈ 2 values, while 4 < g_⊥_ < 6 and g_||_ ≈ 2 values correspond to a QS state,^55,56^ there are several examples in the literature of both HS and QS states featuring g_⊥_ values lower than 6 and 4, respectively.^57^ Further, the pattern of EPR signals displayed by Fe­(III)-Lys9Dab-MC6*a is reminiscent of those reported by Smulevich et al. for the barley peroxidase.^57^ This protein, at pH = 6.5, displays three signals (g = 5.36, 3.75, and 1.93) in a similar spectral region, indicating that distorted admixed-spin systems of this type can yield overlapping HS and QS contributions with apparent g_⊥_ values below 6. Thus, the EPR simulation results are consistent with the Mössbauer (HS+QS) assignment, resolving any apparent ambiguity. The species displaying the slightly higher quadrupole splittings (1.47 and 1.49 mm/s for **1** and **2**, respectively) and the apparent g_⊥_ values below 6 (observed at ∼5.63) may therefore be described either as a highly distorted HS six-coordinate Fe­(III) or as a QS state with a prevalent HS contribution, as confirmed by the simulation-derived parameters reported in Table S1.

The Mössbauer spectra of both isomers in solution, at pH 6.5, feature the signatures of two iron species in ca. 80:20 ratio. The isomer shifts of the most abundant spin entities are identical (0.42 mm/s for both isomers **1** and **2**) whereas the quadrupole splitting values are slightly different (0.97 and 1.14 mm/s for **1** and **2**, respectively; Figure 3d–e green line). The isomer shifts are characteristic of HS heme proteins, whether they are five- or six-coordinate HS. Typical Mössbauer parameters for five-coordinate HS Fe­(III) complexes are ΔE_Q_ = 0.4–1.0 mm/s; δ = 0.25–0.43 mm/s, while for six-coordinate HS ferric complexes, they are ΔE_Q_ = 1.22–2.07 mm/s; δΔ = 0.32–0.45 mm/s.^48,58,59^ The quadrupole splitting values of both isomers, when compared with those reported for ferric heme proteins, particularly fluoro-met-myoglobin and aquo-met-myoglobin,^59^ are indicative of six-coordinate HS ferric porphyrin complexes. On the contrary, the less abundant species display much larger quadrupole splittings (ΔE_Q_ = 2.64 and 3.43 mm/s for isomers **1** and **2**, respectively; Figure 3d-e, blue line) and are indicative of QS spin states. The ΔE_Q_ values of QS states observed for the two isomers are indicative of a different contribution of the S = 5/2 spin state between isomers **1** and **2**. A higher 5/2 than 3/2 spin character in the QS configuration must be ascribed to isomer **1** with lower quadrupole splitting. Overall, based on Mössbauer and pH titration data (see Supporting Information), the prevalent species at pH 6.5 may be assigned to a six-coordinate high-spin Fe­(III)-porphyrin with His-H_2_O axial ligands, whereas the species with an observed 20% relative intensity may correspond to an iron­(III) porphyrin in a QS admixed-spin state, with His-OH^–^ axial ligands.

These findings are also confirmed by EPR data. In fact, both isomer **1** and isomer **2** show very similar EPR spectra with simulated g values at 5.96, 5.50, 2.00, and 5.93, 5.48, and 2.00 (g_*x*_,g_*y*_,g_*z*_) (Figure 3f). These features are consistent with a single HS species (E/D = 0.01) dominating the solution spectra for both isomers, as confirmed by EasySpin simulations (Table S1, Figure S5 in the Supporting Information). The absence of a resolvable QS signal in the solution EPR spectra is consistent with the almost 2 orders of magnitude lower sample concentration used for EPR relative to Mössbauer measurements, which effectively reduces the minor QS component (20% by Mössbauer) below the detection threshold.

In addition, the combined results from Mössbauer and EPR spectroscopy may indicate a concentration-dependent HS:QS distribution. In fact, the relative population of the QS species decreases from ca. 50% in the solid state to ca. 20% in frozen solution samples (in Mössbauer spectra) and becomes undetectable in the more diluted samples used for EPR measurements. Although this interpretation cannot be proven unambiguously on the basis of the present data, it is consistent with a concentration-dependent equilibrium favoring the HS state upon dilution. Such a scenario would reconcile the apparent discrepancy between Mössbauer and EPR data in solution and further support the view that HS and QS states are closely related species whose relative populations are sensitive to the local environment and sample conditions.

### Co­(III)-Lys9Dab-MC6*a: Structure Determination

#### Purification and Preliminary Spectroscopic Analyses

In order to gain structural information from NMR analysis, we prepared the diamagnetic Co­(III) parent derivative of Lys9Dab-MC6*a. The Co­(III) ion was inserted into the apo forms from Co­(II) acetate. Two peaks, named Δ*a*nd Λ, were detected in the reverse-phase HPLC chromatogram for each regioisomer (**1** and **2**) (see Figure S6 in Section S2). The two peaks were then isolated by preparative HPLC and separately analyzed. The ESI-MS analysis revealed the same molecular weight of 3466.9 Da for both species, as expected for Co­(III)-Lys9Dab-MC6*a.

Both species from each regioisomer were stable and clearly distinguishable even at very acidic conditions (pH < 1), thanks to the strong cobalt-histidine coordination that preserves the sandwich structure.

UV/vis and circular dichroism (CD) spectroscopies were combined to analyze the structural features of Co­(III)-Lys9Dab-MC6*a. The UV/vis spectra of the two Δ and Λ complexes, deriving from each regioisomer, are almost superimposable and show spectral features very similar to those of Co­(III)-MC6*a.^21^ The band shapes and positions are consistent with a low-spin Co­(III) species with a His/H_2_O coordination (Figure S6b–d, Section S2).

At pH 6.0, in H_2_O/TFE solution (60/40, v/v), the CD spectra of all four compounds indicate an α-helical structure, as observed for previous MC6 analogues^20,21^ (Figure S7a in Section S2). More interestingly, they show different Cotton effects (CE) in the Soret region. The two cobalt complexes, corresponding to peaks **1Δ** and **2Δ,** display a strong negative CE (408 nm, Figure S7b in Section S2), whereas the complexes corresponding to peaks **1Λ** and **2Λ** display a strong positive CE (409 nm, Figure S7b in Section S2). Based on these results, and in comparison with the prototype Co­(III)-MC1 behavior, the two species in each regioisomer were assigned to the Δ*a*nd Λ diastereomers.^19^

For each regioisomer, the two diastereomeric forms were separated by reverse-phase HPLC, but unfortunately, only the **Δ** isomers were obtained with purity and yield sufficient for structural characterization by nuclear magnetic resonance (NMR).

### Assignment of NMR Resonances

After HPLC purification, only the Δ isomers were obtained with purity and a sufficient yield for structural characterization by Nuclear Magnetic Resonance (NMR).

The ^1^H spectra of both compounds, **1**Δ and **2**Δ, are very well dispersed in the amide region. The spectra display a single set of narrow resonances for the deuteroporphyrin protons and two sets of resonances for the peptide chains (Figure S8 in Section S3). The intensities of each resonance belonging to deuteroporphyrin protons in the *meso* position, as compared with those of the terminal NHs of the peptide chains (Asp1), are in the expected 1:2 ratio. The elements of the secondary structure for **1**Δ and **2**Δ were first delineated using information provided by NOE data and C_α_H conformational shifts (Figure 4).

**Figure 4:**
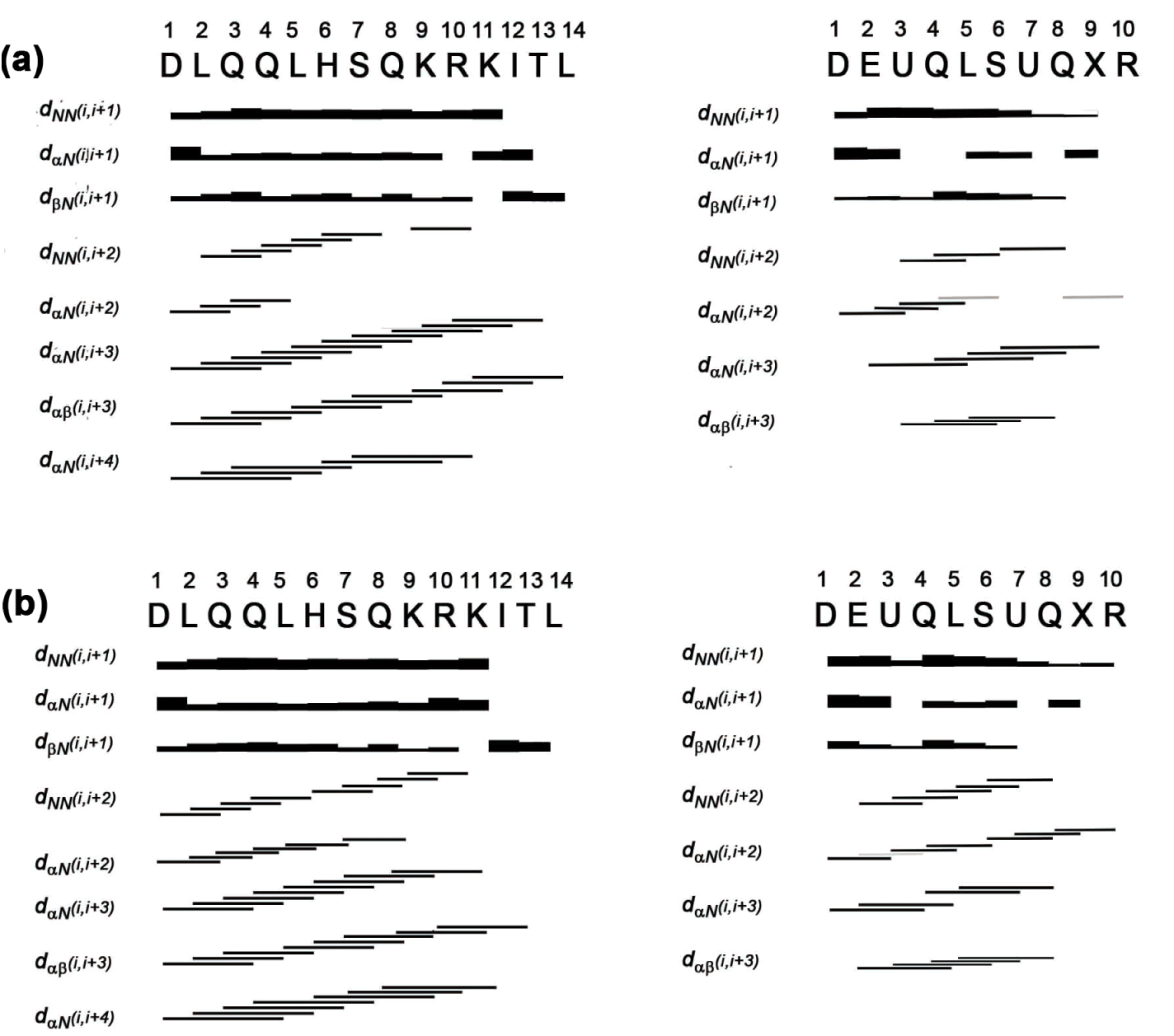
Summary of NOE contacts for (a) Co­(III)-Lys9Dab-MC6*a-**1Δ** and (b) Co­(III)-Lys9Dab-MC6*a-**2Δ**; TD chain (left) and D chain (right). Bar thickness is proportional to the NOE intensity, and gray bars indicate spectral overlap. U and X indicate Aib and Dab residues, respectively.

The two compounds show a very similar pattern of NOE interactions, which strongly supports structural organization for both peptide chains. Specifically, a helical structure can be hypothesized in the regions 1–12 and 3–7 for the TD and D chains, respectively, as confirmed by strong sequential NH–NH as well as medium-range αCH−αCH_(i,i+3)_, NH–NH_(i,i+2)_, αCH–NH_(i,i+3)_ NOE signals. The presence of αCH-NH_(i,i+2)_ connectivities may indicate a partially distorted 3_10_-helical arrangement, which is mainly evident throughout the whole sequence of the D chains in both diastereomers. The helical structure is also supported by the analysis of the Hα chemical shifts.^60^ Notably, almost all residues show upfield-shifted αCH resonances compared to their random coil values (Figure S9 and Table S2 and S3 in Section S3).

Even though both the Δ*a*nd Λ Co­(III)-Lys9Dab-MC6*a complexes are not paramagnetic, several rather unusual chemical shifts have been observed for both side-chain and backbone protons (Table S2 and table S3 in Section S3), reasonably attributable to the deuteroporphyrin ring current.^19^

The protons of the residues Leu2, Leu5, and His6 from the TD chain, and Aib3, Aib7, and Gln8 from the D chain, are positioned right above or below the deuteroporphyrin ring and thus experience considerable upfield shifts with respect to their standard random coil values.^61^ The largest shifts in the side-chain proton resonances are observed for the His6 axial ligand. In particular, the δ-CH and ε-CH imidazole protons are strongly upfield-shifted to −0.20 and −0.45 ppm for **1Δ,** and −0.18 and −0.54 ppm for **2Δ**, respectively (Figure S8c–d, Section S3). It may be noted that these protons exhibit different chemical shifts in the two complexes. This phenomenon suggests a slightly different position of the imidazole rings with respect to the deuteroporphyrin plane. Furthermore, His6 N^δ1^ is protonated for both **1Δ** and **2Δ**, and its proton resonances were well-resolved and found at 9.27 and 9.19 ppm, respectively. These data indicate that the NH^δ1^ protons are not in rapid exchange with the solvent, as is usually observed for imidazole N–H protons, and confirm that *(i)* the Co­(III) axial coordination occurs through the unprotonated imidazole Nε atom, as already found for other Co­(III)-MC complexes, and that *(ii)* His6 residues are buried and inaccessible to solvent.^19^ The designed MC sandwich topology is further confirmed by numerous NOEs between the DPIX protons and the two helical peptide chains (57 and 80 for **1Δ** and **2Δ**, respectively).

### Regioisomer Assignment and Validation of Diastereomer Attribution

The two regioisomers have been provisionally defined as **1** and **2** based only on their HPLC retention time. The presence of NOE contacts between Lys9/Dab9 side chains and the DPIX propionyl groups allowed us to unambiguously identify the positioning of the TD and D chains for the two different regioisomers (Figure 5). Regioisomer **1** is characterized by a set of dipolar contacts along the Dab9 NH^δ^ proton (7.55 ppm), which involves the methylene protons of the propionyl linked at position 2 of the ring (3.10, 3.20, 4.24, and 4.98 ppm). For regioisomer **2**, the set of contacts involving the NH^δ^ proton resonance of Dab9 (7.69 ppm) derives from the propionyl linked at position 18 of the ring (3.14, 3.21, 4.23, and 5.02 ppm).

**Figure 5:**
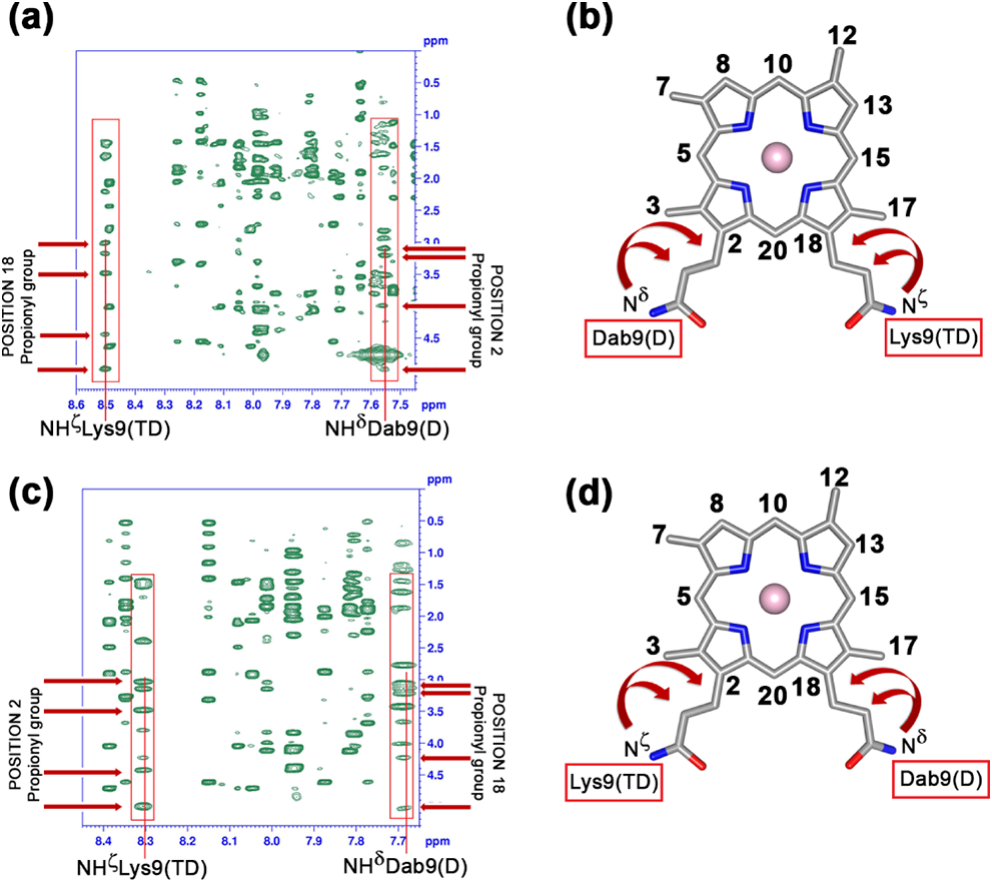
NOESY spectrum regions and schematic representations of the NOE connectivities (arrows) between Lys9/Dab9 amine side chain protons and DPIX propionyl groups at positions 2 and 18 for Co­(III)-Lys9Dab-MC6*a-**1Δ** (a) and (b), and for Co­(III)-Lys9Dab-MC6*a**-2Δ** (c) and (d).

Consequently, TD chains are covalently bound by Lys9 side chains to propionyls at positions 18 and 2 of the ring in regioisomers **1** and **2**, respectively.

To validate the diastereomer tentative attribution based on CD data, we analyzed the dipolar contacts between the TD chain and the DPIX. Among the residues facing the DPIX (Leu2, Leu5, and His6), the most diagnostic contacts come from Leu5. In complex **1**, the Leu5 side chain was involved in dipolar connectivities with 3CH_3_, 5H, 7CH_3_, 8H, and 10H DPIX protons (Figure S10a in Section S3). Since the TD chain of this complex was linked to propionyl at position 18, the presence of these NOE contacts, along with the handedness of the helix, allowed us to position the peptide above the DPIX plane when the porphyrin is seen, as depicted in Figure 5. For complex **2**, the Leu5 side chain was involved in dipolar interactions with 12CH_3_, 13H, 15H, and 17CH_3_ DPIX protons (Figure S10b in Section S3). This finding, together with the right-handedness of the α-helix, indicated that for complex **2,** the TD chain lay below the DPIX plane. Consequently, both complexes were indeed Δ diastereomers.^11,19^

### Structure Calculation and Global Fold Description

We were able to solve the structures of both compounds, obtaining an ensemble of 20 clustered structures. The NMR structures obtained by CYANA^33^ were refined by restrained molecular dynamics (RMD) and then by restrained energy minimization (REM).^37^ The 20 refined structures for **1Δ** (calculated using 15 constraints per residue, including 123 intraresidue, 91 sequential, 74 medium-range, and 70 long-range NOEs) and for **2Δ** (calculated using 18 constraints per residue, including 162 intraresidue, 91 sequential, 106 medium-range, and 87 long-range NOEs) were tightly clustered (Figure 6a–b). The root mean square deviation was 0.84 ± 0.30 and 0.99 ± 0.49 Å for the main chain atoms and 1.88 ± 0.46 and 2.22 ± 0.69 Å for all heavy atoms for **1Δ** and **2Δ**, respectively (Table S4 in Section S3).

**Figure 6:**
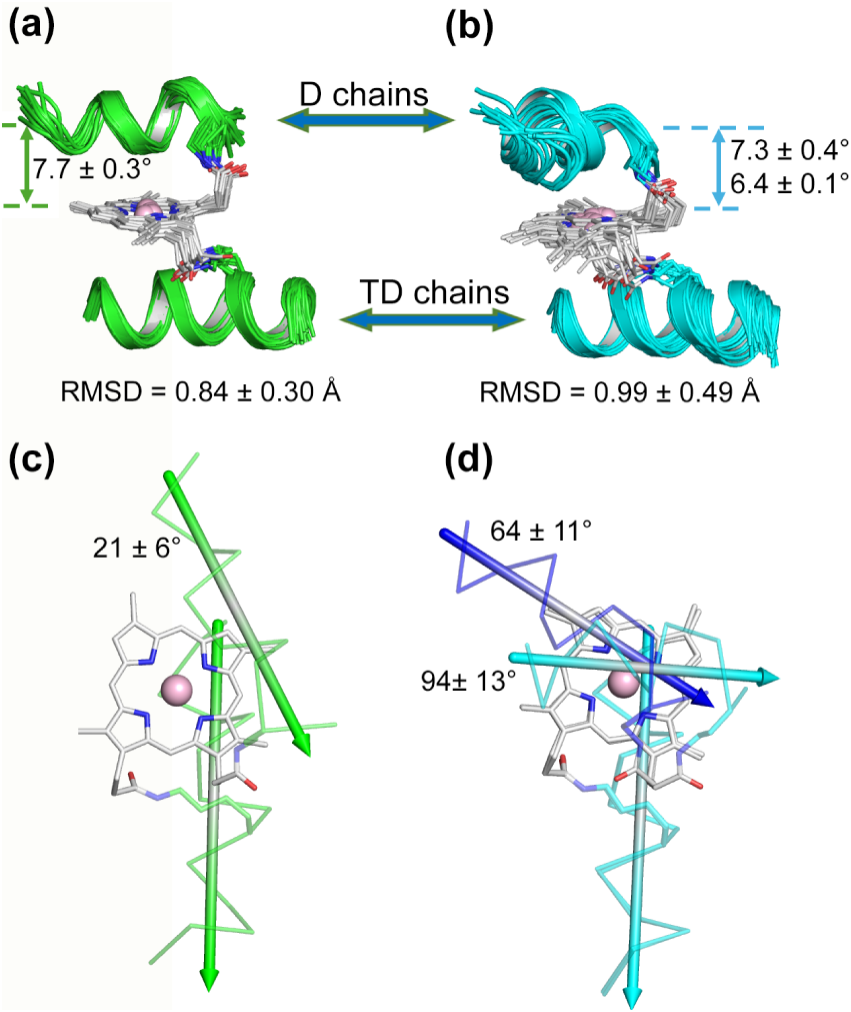
Best backbone superposition of the 20 NMR models of **1Δ** (a, green) and **2Δ** (b, cyan). Peptide chains are shown as cartoons; Dab9, Lys9, and DPIX are shown as sticks, and the cobalt ion is shown as a pink sphere. A top view of **1Δ** (c) and **2Δ** (d) shows that the D chains cover the DPIX to varying extents for the two compounds. Two families can be clustered for **2Δ**, according to the interhelical angles between the D (cyan or blue) and TD (cyan) chains. Colored arrows correspond to the averaged helical axis of the D and TD chains for each family.

The program PROCHECK-NMR^43^ was used to assess the quality of the final NMR ensembles (Table S4 in Section S3). The overall stereochemical quality of the structure ensemble indicates that the structure of **1Δ**Δ is comparable to that of a 1.7 Å well-defined X-ray structure, whereas the structure of **2Δ**Δ compares to a 1.0 Å X-ray structure.

Moreover, all the backbone dihedral angles fall within the most favored and additionally allowed regions of the Ramachandran plot for both isomers (Table S4 in Section S3).

The global fold of **1Δ** and **2Δ** confirms the design hypothesis; both isomers are characterized by a helix-heme-helix sandwich topology, although small differences are observed between the two diastereomers. In particular, while the TD helices lie on the DPIX plane with the same relative orientation for both regioisomers, the D chain is mainly responsible for the higher RMSD of the **2Δ** bundle. For each model, we calculated the interhelical angle, defined as the angle between the TD and D helical axes (Figure 6c,d, and Tables S5 and S6 in Section S4). According to this analysis, the **1Δ** models cluster into a very defined single family featuring an interhelical angle of 21 ± 6°. This makes the helices almost parallel for this regioisomer, resulting in a rather poor coverage of the porphyrin ring distal site, as evidenced by the relatively high solvent-accessible surface area (SASA) of 24 ± 2 Å^2^ on average and by the distance between the Co­(III) ion and the center of mass (CoM) of the D chain backbone (7.7 ± 0.3 Å). On the contrary, the interhelical angle analysis allows us to identify two families of models for the **2Δ** bundle, the former family featuring 12 models and an average angle of 64 ± 11°, and the latter featuring 8 models and an angle of 96 ± 13°. As expected, for both families, the DPIX ring is significantly less exposed to the solvent, the SASA being either 8 ± 3 or 9.2 ± 0.7 Å^2^ for the two families, respectively. The average distance of the cobalt to the CoM of the D chain more clearly conveys the interdependence between the interhelical angle and the distal site coverage in the two sets of models, the former averaging at 7.3 ± 0.4 Å and the latter at 6.4 ± 0.1 Å. Interestingly, the differences between the two structural families may most probably be related to the opposite orientation of the amide bond linking the Dab9 side chain to the propionyl 18 of the DPIX, the carbonyl pointing either inward or outward of the ring for the first and the second family, respectively (Figure S11 in Section S4).

The residues 2–13 of the ″proximal” tetradecapeptide (TD chain) have been found to assume ϕ and ψ torsion angles very close to those expected for a right-handed α-helical conformation in both isomers. The mean values are ϕ = (−68 ± 5)°, ψ = (−36 ± 5)° and ϕ = (−67 ± 8)°, ψΔ= (−36 ± 10)° for the residues Leu2-Thr13 of **1Δ** and **2Δ**, respectively. The residues located in the α-helical region are involved in the typical CO_i_-HN_i+4_ hydrogen bond pattern (from Leu^2^ CO to Leu^14^ NH). A squeezed helical winding is observed at the N- and C-terminal ends, where some residues (Asp1 and Ile12) are involved in CO_i_-HN_i+3_ hydrogen bonds, and acetyl and amide groups are involved in classical helical capping H-bonds. In addition, the Arg10 guanidine group is H-bonded to the propionate carboxyl group in both regioisomers. The helical structure of the TD chain in both isomers brings Leu2, Leu5, and Lys9 side chains to face the DPIX plane (Figure 7a and b). These amino acids form a partially open hydrophobic cage that surrounds the coordinating His6 imidazole ring. Leu2 and Leu5 show a well-defined side chain conformation in both compounds. In **1Δ**, their χ1 and χ2 torsion angles are close to 170° and 60°, respectively, in almost all structures of the NMR bundle (χ1 = −157 ± 6°, χ2= 82 ± 8° for Leu2 and χ1 = −175 ± 3°, χ2 = 64 ± 6° for Leu5, Figure 7a). This combination of side chain dihedral angles corresponds to that observed for the rotamer designated as “tp” in the “penultimate rotamer library” (in a “tp” rotamer, the letter t is for “trans”, χΔ = 180°, and p is for “plus”, χ = +60°),^62^ one of the most common rotamers found for leucine. In **2Δ**, Leu2 and Leu5 adopt different side chain conformations. Leu2 assumes an “mt” conformation (where m is for “minus”, χ = −60°) with χ1 = −80 ± 2° and χ2 = 172 ± 6°, the most common rotamer for Leu,^62^ whereas Leu5 adopts the “tt” rotamer (χ1 = −161 ± 5° and χ2 = 170 ± 4°, Figure 7b). In both complexes, His6 assumes a well-defined side chain conformation: it adopts a single conformer with χ1 = (−76 ± 1)° and χ2 = (86 ± 2)° in **1Δ** and χ1 = (−66 ± 1)° and χ2 = (103 ± 4)° in **2Δ**. The observed side chain conformations are in agreement with χ1 and χ2 populations observed for heme ligand histidines.^16^ Most of the charged side chains (Lys, Arg, Glu, and Asp) are solvent-exposed (Figure 7a,b).

**Figure 7:**
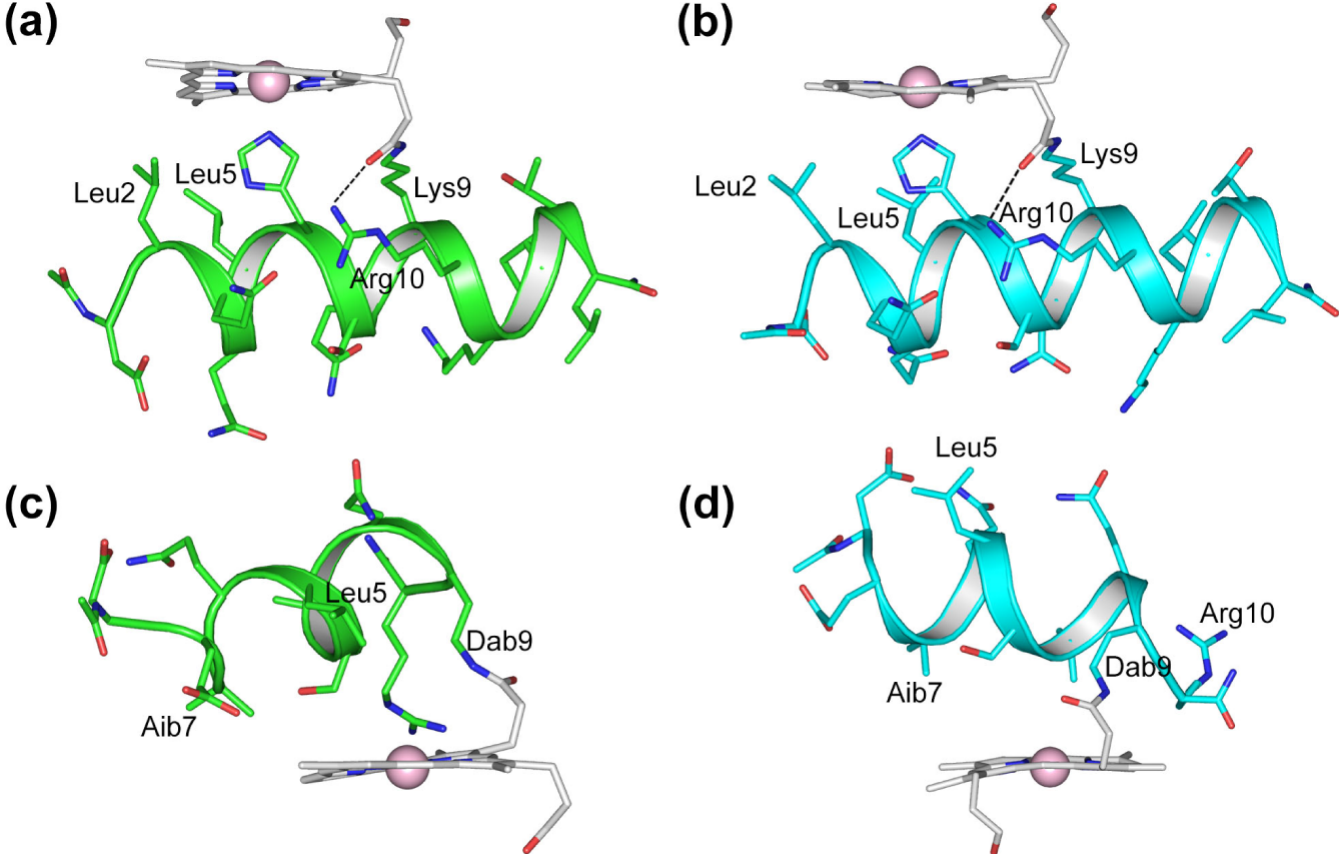
Backbone and side chain conformations of the lowest energy models for TD (a, b) and D (c, d) chains. The **1Δ** diastereomer is shown in green (a, c), and the **2Δ** in cyan (b, d). Peptide backbones are shown as cartoons, side chains and DPIX as sticks, and cobalt as a sphere. Selected hydrogen-bond interactions are reported as dashed lines.

The D chain adopts a less regular α-helical structure with a distortion toward a 3_10_ helix. The mean values for ϕ and ψ angles, calculated for the residues Leu2-Dab9, are ϕ = (−70 ± 15)°, ψ = (−29 ± 12)° and ϕ = (−71 ± 17)° and ψ = (−26 ± 10)°, for **1Δ** and **2Δ**, respectively. In **1Δ**, the helical arrangement of the D chain allows Aib3, Leu5, and Aib7 side chains to face the DPIX plane and interact with the right side of the macrocycle (Figure 7c). As a further consequence, the Leu5 side chain directly points toward the distal site of the metal, making a hydrophobic environment. In most models, Arg10 is involved in an H-bond interaction with the carbonyl of the propionyl group. This results in the amide proton of the Dab9 side chain pointing toward the distal site in 13 out of 20 models. In **2Δ**, the Aib3 and Aib7 side chains are so tightly packed against the upper left side of the macrocycle that Leu5 now points toward the solvent, in the opposite direction of the distal site (Figure 7d). In particular, the Aib7 methyl groups display chemical shift values smaller than their average values reported in the BMRB database,^63^ due to the ring-current shielding effect, as previously observed for Co­(III)-MC6*a.^20^ Consequently, the active site is even more hindered and hydrophobic than **1Δ**, as already highlighted by a SASA comparison. Unlike the other regioisomer, Arg10 in **2Δ**, is involved in C-terminal helix-capping by H-bonding the carbonyl of Aib7 (11/20 models).

### Histidine Orientation

In both complexes, the plane of the His6 imidazole ring is almost perpendicular to that of the DPIX ring, but the orientation of the imidazole ring with respect to the deuteroporphyrin plane is different. This structural feature is one of the most noticeable differences between the **1Δ** and **2Δ**, despite the almost identical structural arrangement of the TD chains, and it was established thanks to the connectivities between His6 δ-CH and ε-CH protons and the deuteroporphyrin protons.

Specifically, for **1Δ,** the following NOE contacts were found: εCH–7CH_3_, ΔεCH–8H, δCH–17CH_3_, δCH–15H. For compound **2Δ**, we found the following dipolar contacts: εCH–12CH_3_, εCH–10H, δCH–3CH_3_, δCH–20H. These connectivities indicated that the imidazole planes intersected the DPIX ring along its 9–19 and 10–20 positions for **1Δ** and **2Δ**, respectively. Moreover, several dipolar contacts have been observed between His6 and a set of residues of the TD chain (Leu2, Gln3, Leu5, and Ser7), supporting the univocal orientation of the imidazole ring for both complexes.

Structural calculations confirmed that the imidazole ring is oriented along the 9 and 19 positions in **1Δ**, nearly eclipsing the N23–Co–N24 bond, while it is positioned close to the 10 and 20 positions (α and γ meso positions) in **2Δ**. The angle between the reference axis, N21–N23, and the His plane normal, θ, for **1Δ** and **2ΔΔ** is −33.4° and 35.1°, respectively (Figure 8).

**Figure 8:**
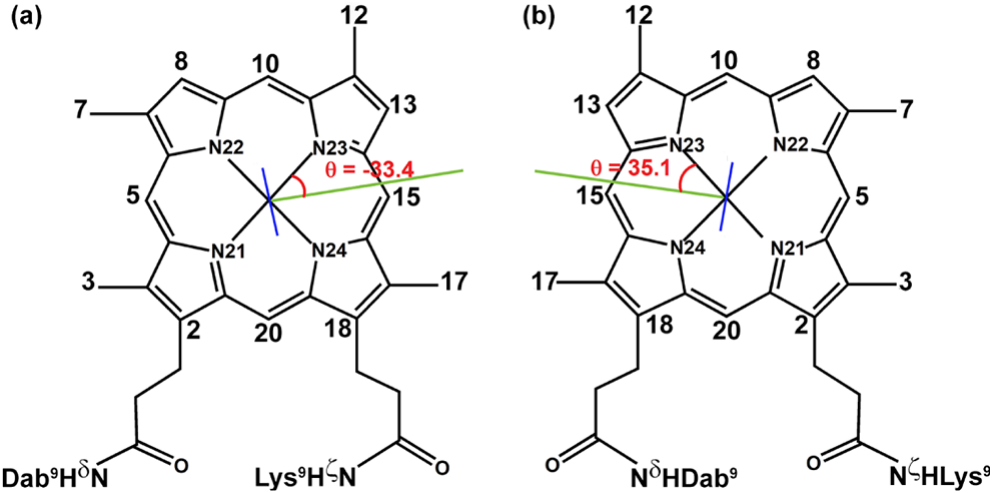
Schematic representation of the DPIX moiety and histidine axial ligand in Co­(III)-Lys9DabMC6*a-**1Δ** (a) and Co­(III)-Lys9DabMC6*a-**2Δ** (b). The reference axis is taken along the N21–N23 direction. The θ angle defines the acute angle between the reference axis and the His plane normal (green line). Structures are projected to have the imidazoles (blue lines) binding from the viewer’s perspective.

### Functional Implications

Axial ligands and their bonding geometry, as well as the metal electronic state, are crucial in modulating the properties of the iron-porphyrin cofactor, as demonstrated by theoretical and experimental studies carried out on heme protein models.^7,64,65^ It is widely accepted that the orientation of the histidine, axially ligated to heme, can strongly influence the function of heme cofactors in proteins^66^ and its different positioning may finely tune reactivity and produce functional diversity.^23^ The θ values observed in **1Δ** and **2Δ** Co­(III) complexes fairly compare with those calculated for the Fe­(III)-Lys9DabMC6*a complexes, within ±10° (Table S7 in Section S4).^23^

This small divergence may be ascribed to the presence of cyanide in the Fe­(III) complexes, a very strong-field exogenous sixth ligand, needed to decrease the spin and enable the paramagnetic analysis.^23^ As already observed for natural peroxidases, cyanide binding induces some rearrangements of residues around the heme moiety and may generate differences not only in the spin-density distribution of the heme but also in the structure of the active site. For example, it was already shown that the orientation of the histidine axial ligand is quite different in the cyanide-derivative of lignin peroxidase (LiP-CN^–^), as compared to the uncomplexed form of the enzyme.^67^ Nevertheless, the different histidine orientations observed for the two complexes may have a bearing on their catalytic and electronic properties, with Fe­(III)-Lys9DabMC6*a-**2** showing a higher turnover frequency (*k*_cat_) compared to the other isomer.^23^

Second-sphere interactions play vital roles in modulating the reactivity of heme proteins and are deemed important in naturally occurring heme active sites for substrate recognition and catalysis. By extending the structural characterization of the cobalt complex to the iron one, we can show that not only are mimochromes able to deliver sufficient information in their short peptide sequences to impart function, but also that the two regioisomers display remarkably different second-sphere interactions despite sharing the same peptide composition. The different rotamers observed in the hydrophobic pocket around the coordinating His6 in the **1Δ** and **2Δ** isomers affect the side chain-main chain hydrogen bond of His6. Surprisingly, while in **1Δ** the His6 δ1-NH group forms H-bonds with the Leu2 CO group in all the refined models, the same does not hold true in **2Δ**, for which a satisfactory distance but unideal angle geometry was found (Figure 9 and Table S8). This H-bond represents an important structural feature in several natural hemeproteins.^68^ A stronger H-bond increases the σ-donation to the metal by the imidazole, thus stabilizing higher redox states and lowering the redox potential. This finding has two important consequences: *(i)* out-of-plane Fe­(III) distortion may be responsible for the increased HS character of the QS spin state in **1Δ**; (ii) the redox potential of the Fe­(IV)=O|Fe­(III) couple is expected to be lower, negatively impacting the thermodynamic driving force for substrate oxidation in **1Δ**. Nevertheless, structural analyses have highlighted the increased accessibility at the distal site for **1Δ**, thanks to a more “open” orientation of the D chain. Interestingly, **1Δ** displays a 10-times lower *K*_m_ versus ABTS,^23^ as expected for a preorganized substrate-binding pocket. To further corroborate this hypothesis, a remarkable difference in terms of H-bonds between **1Δ** and **2Δ**, is found in the distal cavity. In particular, while in **1Δ** the Ser6 (D chain) is hydrogen-bonded to the Aib3 carbonyl backbone and to the hydroxide molecule bonded to Co­(III) (W1), with an occurrence of 75% and 25%, respectively, in **2Δ** the same residue forms only a hydrogen bond with the Glu2 CO (Figure 9). This finding is not completely unexpected and may represent a further hint to explain the different reactivity found for the two compounds. While helping in orienting the substrate, this set of interactions may be detrimental to the TOF of **1Δ.**

**Figure 9:**
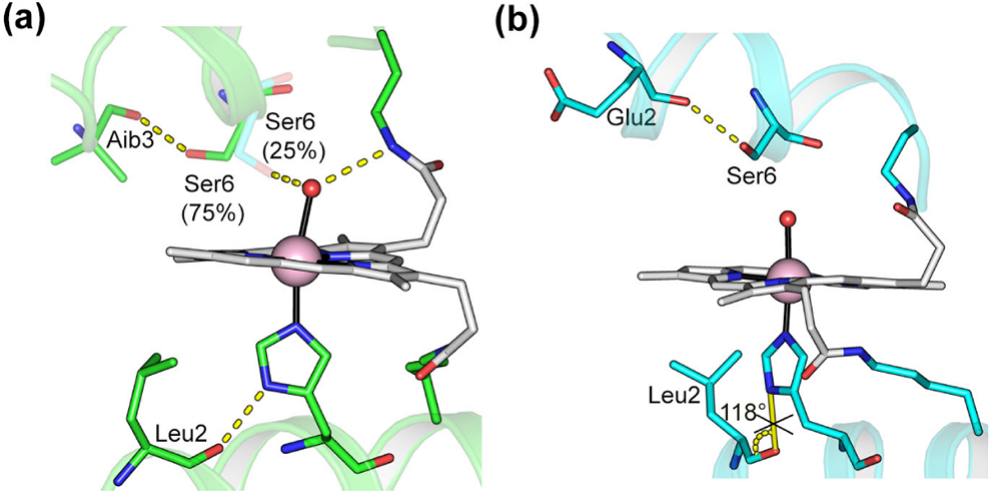
Second sphere interactions for **1Δ** (a) and **2Δ** (b).

We have demonstrated that the catalytic cycle of iron-bound MC analogues occurs via a peroxidase-like mechanism through the formation of high-valent oxo-ferryl species (Cpd I). An important question concerning the catalytic cycles of enzymes is the mechanism of formation of the active species. It has been found that, in peroxidases, the highly oxidized intermediate is generated by a transient ferric hydroperoxide (Cpd 0) intermediate, obtained by the reaction of the resting Fe­(III) state with hydrogen peroxide.^7^ Protonation of the distal oxygen of the hydroperoxide complex is necessary for the conversion of Cpd 0 into Cpd I, facilitated by residues in the distal pocket acting as acid–base catalysts.^8^ We may speculate that the lower TOF observed for compound **1Δ** could be inferred to the presence of a network of non acidic H-bond donors from both the Ser6 alcohol moiety and the Dab9 amide bound to the DPIX propionyl group. These two interactions may help in stabilizing the Cpd 0, affecting the O–O bond cleavage and consequently can reduce the formation of Cpd I. Conversely, the more hindered distal pocket of **2Δ**, though less efficient in substrate binding, favors the protonation of the distal oxygen of the hydroperoxo intermediate, where both Ser6 and the carbonyl from the DPIX can most probably stabilize a network of water molecules that perform the required protonation toward Cpd I, as already suggested for small-molecule catalysts.^7^

## Conclusion

In this work, a thorough characterization of an MC6 analogue has been performed on both the Fe­(III) and Co­(III) complexes by^57^Fe Mössbauer, EPR and NMR spectroscopies. The adoption of a shorter linker for the distal peptide enabled us to thoroughly characterize both the isolated regioisomeric and diastereomeric species. We previously demonstrated that, in an aqueous solution containing >40% TFE, the **Δ** diastereomers are favored for the fast-exchanging iron complex. This simplified the spectroscopic characterization and, most prominently, led to the definition of the spin state for the two Fe-**1Δ** and Fe-**2Δ** regioisomers. Whereas, when cobalt is bound, both diastereomers can be trapped. Unfortunately, only two out of four of them could be isolated in sufficient purity/amount for structural characterization. Nevertheless, we serendipitously found that the two isolated species were, in fact, Co-**1Δ**Δ and Co-**2ΔΔ** regioisomers, enabling us to directly correlate the structural analysis to both catalytic and electronic studies. Thanks to the available structural information, we demonstrated, for the first time in the MC6 class, that both TD and D chains help in shaping the metal site activity and the binding site accessibility, recapitulating several hallmarks of natural proteins in a ∼3 kDa metalloenzyme. Moreover, we show that a difference as small as a methyl group on the DPIX is sufficient in determining a dramatic shift in the catalytic features of the two regioisomers. This helped us in finding a rationale for the peculiar trend found for the two regioisomers, where the first (**1Δ**) featured a better *K*_m_ but a worse *k*_cat_, while exactly the opposite was found for the second regioisomer (**2Δ**). Thanks to this new starting point, structurally guided designs will be carried out by focusing on the well-established hydrogen evolution activity of the cobalt Mimochromes.^21,22^

## Supplementary Material



## Data Availability

The parametrized DPIX (with the amidated propionic groups), Aib residue, Dab, and Lys residue acetylated on the side chain were obtained using Antechamber,^39^ and the computational data are available in a Zenodo repository (https://zenodo.org/records/17019610).

## Notes

Additional experimental details; spectroscopic data (UV/vis titrations, Mössbauer and EPR analysis, CD analysis); structural characterization by NMR (with pertinent figures); additional tables (Mössbauer and EPR parameters; ^1^H chemical shift; NMR and refinement statistics; calculated structural parameters, H-bonds); and additional figures (PDF)

## ∥

Merck Serono, Via L. Einaudi, 11, 00012 Guidonia Montecelio (RM), Italy

## ⊥

BBI Solutions, Unit 2 Parkway, Pen-y-fan Industrial Estate, Crumlin, NP11 3EF, UK

## Abbreviation

TFA: Trifluoroacetic acid
TFE: 2,2,2 Trifluoroethanol
NMR: Nuclear Magnetic Resonance.
EPR: Electron Paramagnetic Resonance
NOESY: Nuclear Overhauser Effect SpectroscopY
TOCSY: TOtal Correlation SpectroscopY
DQF-COSY: Double Quantum Filtered COrrelation SpectroscopY
PDB: Protein Data Bank
HS: high spin
LS: low spin
QS: quantum mechanical admixed-spin
