# Supplementary material for: Iron to Cobalt Swapping in a Bioinspired Heme-Peroxidase: Structural Characterization and Functional Implications
Source: Inorg Chem. 2026 Apr 23;65(17):9359–71. doi: 10.1021/acs.inorgchem.5c05553 (PMC13147336; doi:10.1021/acs.inorgchem.5c05553)
Supplement: Supplementary file 1 [file ic5c05553_si_001.pdf]

Supplementary Information

Iron to cobalt swapping in a bioinspired heme-peroxidase: structural characterization and functional implications

Marco Chino,[†] Corinne Cerrone,[†] Fabio Pirro,[†] Maria De Fenza,[†] Serhiy Demeshko,[‡] Ornella Maglio,[†,§] Franc Meyer[‡] and Angela Lombardi[†,*]*

† Department of Chemical Sciences, University of Naples Federico II, Via Cintia 21, Napoli 80126, Italy.

§ Institute of Biostructures and Bioimaging (IBB), National Research Council (CNR), Via P. Castellino, Napoli 80131, Italy.

‡ Institute of Inorganic Chemistry, University of Göttingen, Tammannstraße 4, Göttingen 37077, Germany.

Corresponding Authors

Angela Lombardi, email: alombard@unina.it; Ornella Maglio, email: ornella.maglio@unina.it

Table of Contents

Figure S1	Deuteroporphyrin chemical structure	S3
Section S1	Fe(III)-Lys9Dab-MC6*a-**1** and **2:** Synthesis and spectroscopic characterization<br>Figures S2, S3, S4 and S5<br>Table S1	S4
Section S2	Co(III)-Lys9Dab-MC6*a-**1** and **2:** Synthesis and spectroscopic characterization<br>Figures S6 and S7	S9
Section S3	Structure determination of Co(III)-Lys9Dab-MC6*a-**1Δ** and **2Δ** by NMR<br>Figures S8, S9 and S10<br>Tables S2, S3 and S4	S12
Section S4	Computational details for structural analysis of Co(III)-Lys9Dab-MC6*a-**1Δ** and **2Δ**<br>Figure S11<br>Tables S5, S6, S7 and S8	S22
Section S5	References	S29

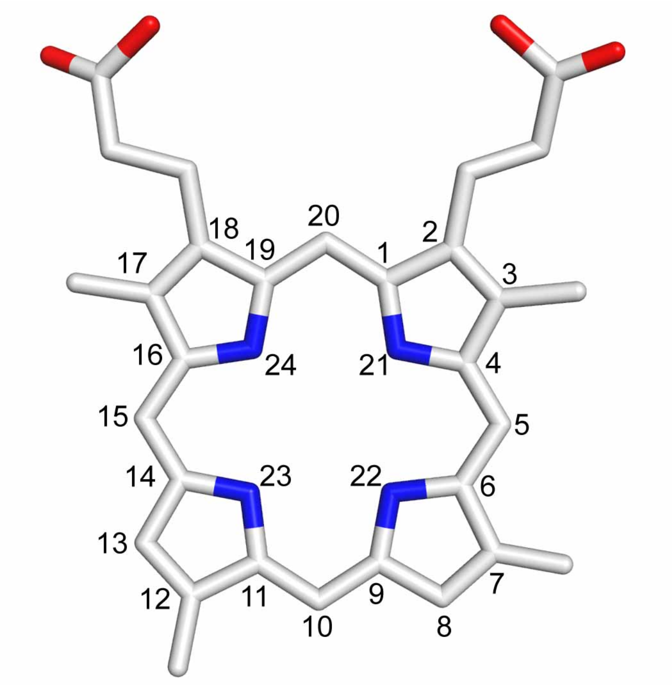

Figure S1. Schematic representation, depicting the deuteroporphyrin chemical structure. The numbering scheme according to the IUPAC nomenclature is also reported; commonly, the position 5, 10, 15 and 20 have been also referred to as meso-positions.

Section S1: Fe(III)-Lys9Dab-MC6*a-1 and 2

✓ *Synthesis and purification*

Fe-Lys9Dab-MC6*a was synthesized and purified according to previously reported procedures.[1] Stock solutions were prepared by dissolving the lyophilized compound in H_2O 0.1% TFA (*v/v*) and were stored at -20 °C until their use. Fe-Lys9DabMC6*a-**1** and Fe-[1]Lys9DabMC6*a-**2** concentrations were determined by UV/Vis spectroscopy, using ε (389) = 1.50 $\times 10^5$ $M^{-1}cm^{-1}$, and 1.80 $\times 10^5$ $M^{-1}cm^{-1}$ (H_2O, 0.1% TFA *v/v*), respectively.[1]

✓ *UV/Vis pH titration of Fe(III)- Lys9Dab-MC6*a-**1** and **2***

The coordination properties of Fe(III)-Lys9Dab-MC6*a-**1** and Fe(III)-Lys9Dab-MC6*a-**2** were investigated by UV-vis pH titration, following the absorbance modifications in the 2.0-9.0 pH range, in a TFE–water solution (50% *v/v*) (Figure S2 and S3) The molar extinction coefficient (ε) at 389 nm was plotted as a function of pH. Data points were fitted has previously reported.[2]

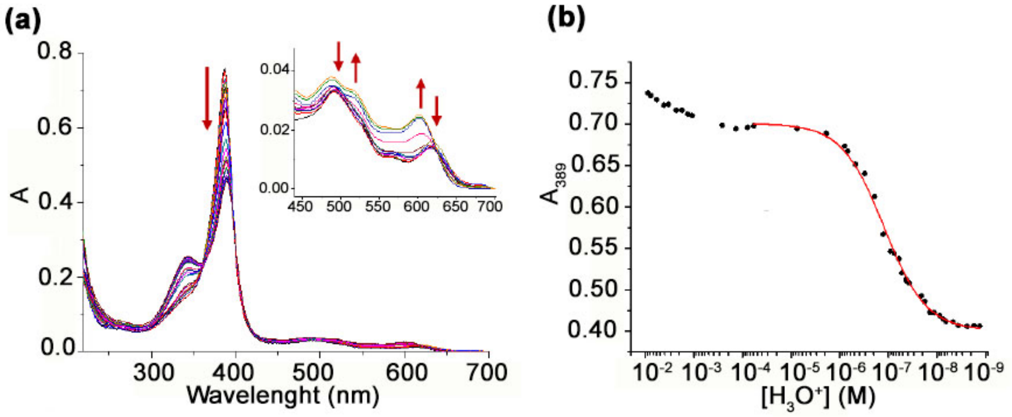

Figure S2. Coordination properties of Fe(III)-Lys9Dab-MC6*a-**1**. a) UV/Vis-pH titration of Fe(III)-Lys9Dab-MC6*a-**1** (5.0 10^{-6}M) in H_2O/TFE solution (50% *v/v*). The spectroscopic titration was performed using NaOH (at concentrations in the range 0.1-1M), in a 1.0 cm path length cuvette. Arrows indicate changes of the Soret-band from acidic to alkaline pH. b) Plot of the Soret absorbance at 389 nm against pH, showing a picture of the species (see arrows) involved in the pH-dependent equilibria. Data points were fitted as previously reported.[2]

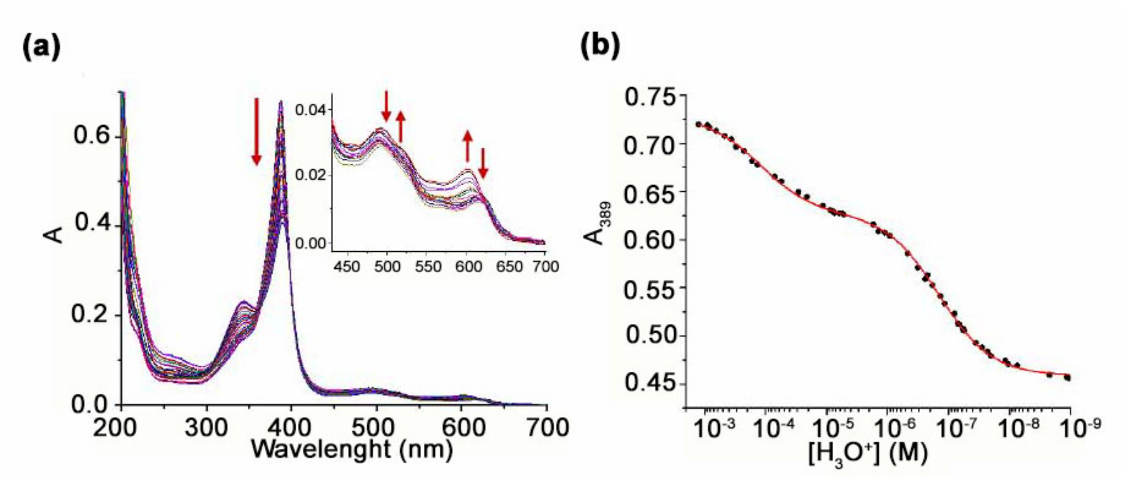

Figure S3. Coordination properties of Fe(III)-Lys9Dab-MC6*a-**2**. a) UV/Vis-pH titration of Fe(III)-Lys9Dab-MC6*a-**2** (6.0 10^{-6}M) in H_2O/TFE solution (50% *v/v*). The spectroscopic titration was performed using NaOH (at concentrations in the range 0.1-1.0 M), in a 1.0 cm path length cuvette. Arrows indicate changes of the Soret- band from acidic to alkaline pH. b) Plot of the Soret absorbance at 389 nm against pH, showing a picture of the species (see arrows) involved in the pH-dependent equilibria. Data points were fitted as previously reported.[2]

For Fe(III)-Lys9Dab-MC6*a-**1**, one well-resolved transition with midpoint at pH 6.9 ± 0.1 was observed. It was not possible to determine the pKa in the pH range 2.0-5.0, because at pH 2.0 the plateau is still not reached (see Figure S2b). The spectrum at this pH value can be attributed to a mixture of mainly high-spin bis-H_2O and His-H_2O ferric complexes.

At pH 5.0, the Soret bands is at 388 nm, the Q bands at 493 and 530 nm, and the charge-tranfer (CT) band at 618 nm: these observations are consistent with a His/H_2O ferric ion axial coordination, predominantly in high spin state.[3,4]

At pH around 8.0, the Soret band shifts to 389 nm, the Q band is observed as a shoulder at 514 nm; the main bands at 487 and 600 nm are attributed to the CT bands of the mainly high-spin ferric hydroxide complex.[5,6] These spectroscopic features suggest that the transition with midpoint at pH 6.93 corresponds to a ligand exchange from H_2O to OH^-.

For Fe(III)-Lys9Dab-MC6*a-**2**, two well-resolved transitions were observed, with midpoint at pH 3.9 ± 0.1 and 6.8 ± 0.1 (Figure S3b). At pH around 3.0, the spectrum exhibits the Soret band at 387 nm, the Q bands at 491 and 528 nm, and the CT band at 614 nm. These spectral features are

representative of a 6-coordinate His-H_2O species, mainly in the HS state. An increase in the pH from 3.0 to 5.3 causes a decrease in intensity and a slight red shift of the Soret (388 nm) and CT bands (615 nm): these observations are consistent with the change in the ferric ion axial coordination from H_2O/H_2O to His/H_2O.[3,4] At pH around 8.0, the Soret band shifts to 390 nm, the Q band appears as a shoulder at 524 nm; the band at 488 and 602 nm can be attributed to the CT band of the ferric complex with the hydroxide ion mainly in the high spin state.[5,6] Therefore, also for Fe(III)-Lys9Dab-MC6*a-**2**, the second transition corresponds to a ligand exchange in the ferric ion axial coordination from H_2O to OH^-.

✓ *Mossbauer and EPR analysis of Fe(III)- Lys9Dab-MC6*a-**1** and **2***

Mossbauer and EPR analyses have been performed as described in the Experimental section of the main text. Here the main fitted parameters are reported for simplicity (Table S1). EPR spectra were simulated for isomers **1** and **2**, both in solid and solution states. Figure S4 shows fitting results only for Fe(III)-Lys9Dab-MC6*a-**1**, for clarity. A representation of the spin states of Fe(III) heme proteins.is schematically shown in Fig. S5.

Table S1. Compared Mössbauer and EPR_parameters for Fe(III)-Lys9Dab-MC6*a isomer **1** and **2**

		**1**<br>Solid state	**2**<br>Solid state	**1**<br>Solution state	**2**<br>Solution state
Mössbauer<br>T = 6 K	δ (mm s^{-1})	0.42; 0.40	0.43; 0.41	0.42;0.37	0.42; 0.39
	ΔE_Q (mm s^{-1})	1.47; 3.20	1.49; 3.21	0.97; 2.64	1.14; 3.43
	FWHM (mm s^{-1})	0.43; 0.48	0.46; 0.45	0.38; 0.32	0.34; 0.33
	Relative Intensity (%)	53; 47	53; 47	81; 19	82; 18
	Assigned Spin State	HS; QS	HS; QS	HS; QS	HS; QS
EPR<br>T = 4 K	g_x/g_y	5.92/5.58;<br>5.92/3.56	5.92/5.58;<br>5.92/3.56	5.96/5.50	5.93/5.48
	g_z	2.00	2.00	2.00	2.00
	Relative Intensity (%)	82; 18	80; 20		
	E/D	0.01; 0.04	0.01; 0.04	0.01	0.01
	Assigned Spin State	HS; QS	HS; QS	HS	HS

Figure S4. EPR spectra of Fe(III)-Lys[9]Dab-MC6*a-**1** in solid (a) and solution (b) states. Acquired spectra are reported as solid grey lines, baseline-corrected spectra are reported as black solid lines, fitted spectra are reported as olive solid lines while spectrum components for solid state are reported as dashed lines.

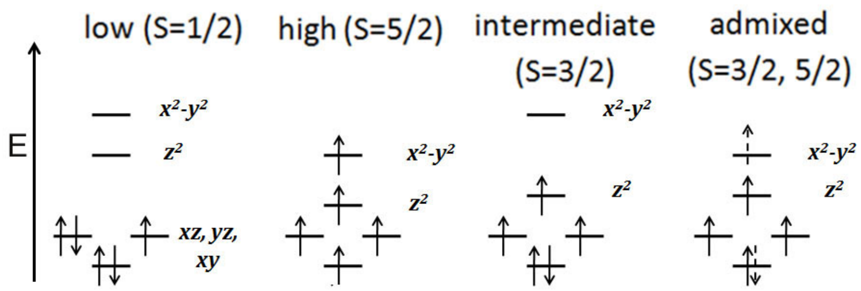

Figure S4. Schematic distribution of d-orbitals giving rise to different electronic spin ground states in Fe(III) heme proteins.

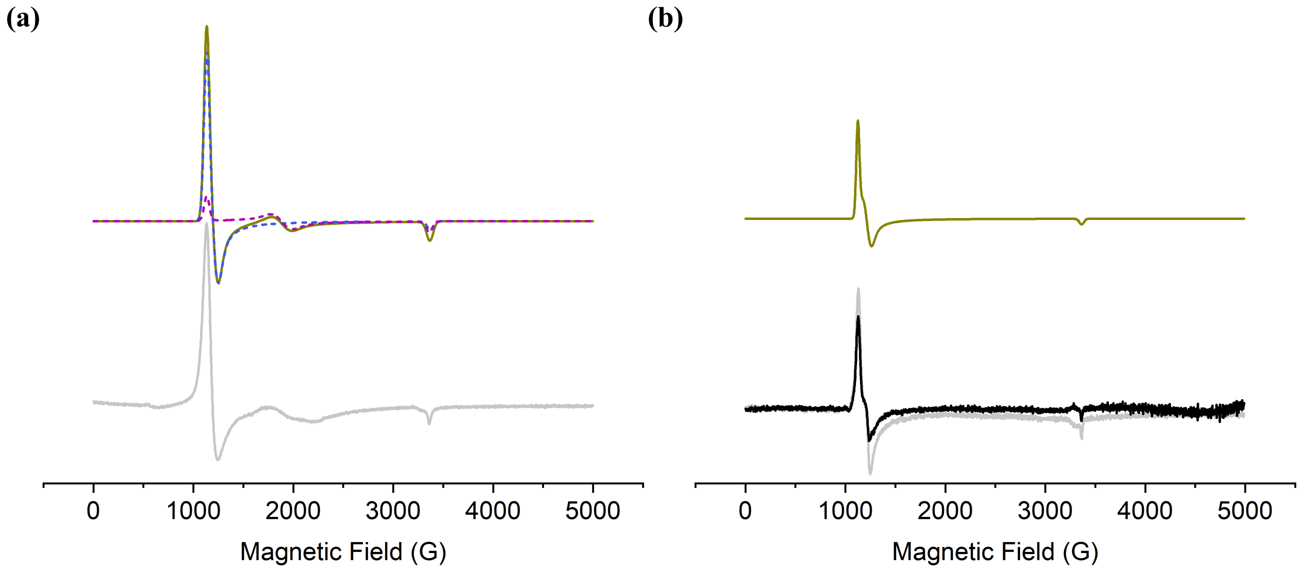

Figure S5. EPR spectra of Fe(III)-Lys^{9}Dab-MC6*a-**1** in solid (a) and solution (b) states. Acquired spectra are reported as solid grey lines, baseline-corrected spectra are reported as black solid lines, fitted spectra are reported as olive solid lines while spectrum components for solid state are reported as dashed lines.

Section S2: Co(III)-Lys9Dab-MC6*a-1 and 2

✓ *Cobalt insertion into Lys9Dab-MC6*a and UV-Vis characterization*

Cobalt ion was inserted into Lys9Dab-MC6*a free bases regioisomer **1** and regioisomer **2**, according to the acetate method procedure slightly modified by us.[7–9]

Co(II) acetate (10 eq.) was added to a solution of both pure apo forms ($2.0 \cdot 10^{-4}$ M), in an acetic acid/TFE mixture (3:2 *v/v*), and the reaction mixtures were kept at 50 °C for 2 h, under reflux.

The reactions were monitored by analytical RP-HPLC using a C18 column (4.6 mm·150 mm; 5 µm) eluted with a linear gradient of acetonitrile in aqueous 0.1% TFA (*v/v*), from 10% to 50% over30 min, at 1.0 $mL \cdot min^{-1}$ flow rate.

For both reactions, two species were found in about the same ratio (Figure S6a and c).

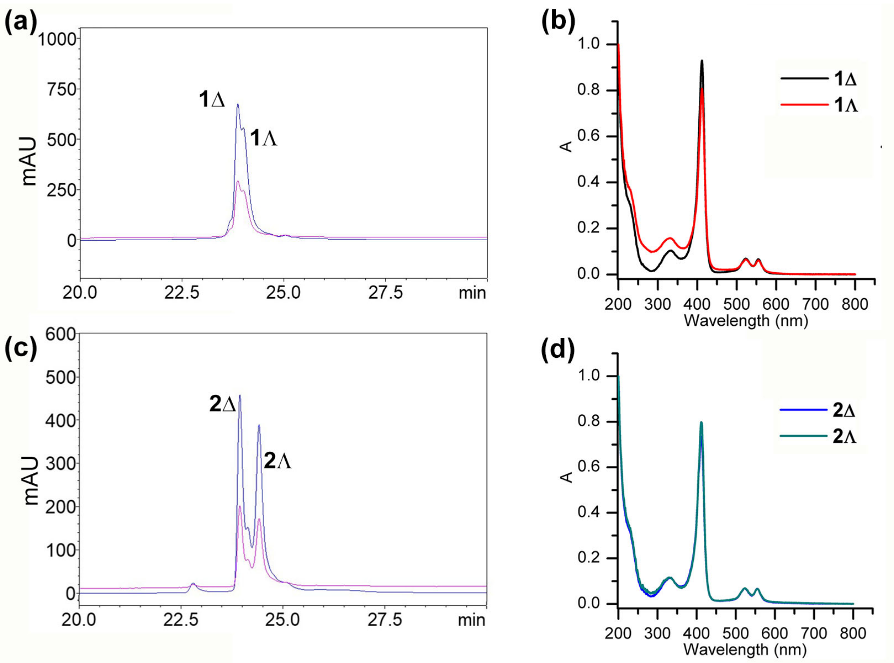

Figure S6. RP-HPLC chromatograms of (a) Co(III)-Lys[9]Dab-MC6*a-**1** and (c) Co(III)-Lys[9]Dab-MC6*a-**2** (λ=210 nm, magenta line; λ=412 nm, cyano line). Uv-vis spectra of (b) Co(III)-Lys[9]Dab-MC6*a **1Δ** and **1Λ** isomers and of (d) **2Δ** and **2Λ** isomers in H_2O/TFE (40%, *v/v*) pH = 6.0. Position and intensities of the Soret: band; and the β and α bands are characteristic of an octahedral Co(III) complex for all isomers.

Once the reaction was completed, the solvent was removed under vacuum and the products were purified from the excess of cobalt acetate by Reverse Phase-Flash Chromatography, on a SNAP KP-C18-HS 30 g column, using a gradient of acetonitrile in 0.1% aqueous TFA, 5% to 95% over 2 column volumes, at 25 $mL \cdot min^{-1}$ flow rate.

The two species were then separated by preparative RP-HPLC and the analysis by ESI-MS confirmed the purity of the products and revealed that all four compounds have a molecular weight, of 3466.9 Da, in agreement with the mass expected for Co(III)-Lys9Dab-MC6*a.

Spectral analysis was then performed on all cobalt-containing isomers.

✓ *CD analysis*

CD spectra in the far-UV region and in the CD spectra in the Soret region of the four compounds, obtained after cobalt insertion into Lys9Dab-MC6*a free bases regioisomer **1** and regioisomer **2**, were performed at pH 6.0 in presence of 40 % TFE (*v/v*) (Figure S7). The four compounds are characterized by Cotton effects of different signs, thus indicating that both regioisomers occur as two diastereomeric forms: Δ and Λ.

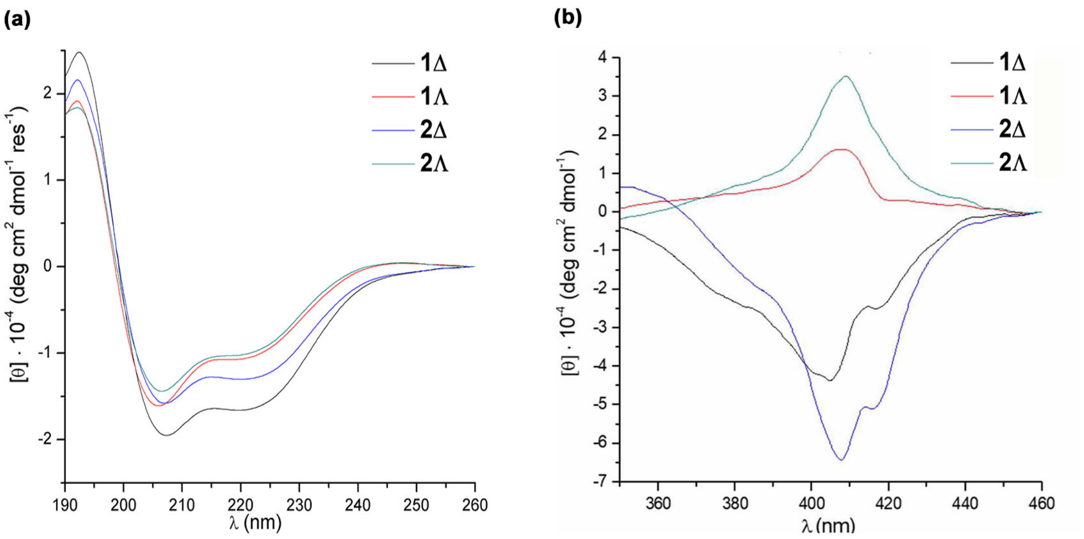

Figure S7. CD spectra in the far UV region (a) and in the Soret region (b) of Co(III)-Lys9Dab-MC6*a isomers in H_2O/TFE (40%, *v/v*) pH = 6.0.

Section S3: Structure determination of Co(III)-Lys9Dab-MC6*a-1Δ and 2Δ by NMR

The two Co(III)-Lys9Dab-MC6*a complexes, purified by preparative RP-HPLC, were separately analyzed by Nuclear Magnetic Resonance. The analysis was carried out in H_2O/TFE 60/40 (*v/v*).

The ^1H chemical shifts (in ppm) of Co(III)-Lys9Dab-MC6*a-**1Δ** and **2Δ** are reported in Table S2 and S3, respectively. The elements of secondary structure were determined on the basis of the chemical shift index (CSI)[10] and of the NOEs.

Table S2a: Co(III)-Lys9Dab-MC6*a-**1Δ** ^{1}H chemical shift (ppm)

Tetradecapeptide (TD chain)						
Residue	NH	$C_\alpha H$	$C_\beta H$	$C_\gamma H$	$C_\delta H$	Others
Ac						1.98
Asp[1]	7.73	4.55	2.71			
Leu[2]	8.18	3.34	1.42, 1.24	0.47	0.98, 0.68	
Gln[3]	8.25	3.29	1.90, 1.83	2.05		7.18, 6.49 (ε)
Gln[4]	7.98	3.68	1.90	2.28, 2.19		7.27, 6.55 (ε)
Leu[5]	7.63	3.54	1.06, 0.53	0.79	0.59, 0.01	
His[6]	6.33	2.30	1.48, 1.12			9.27 (δ1),-0.20, (ε1)<br>-0.45 (δ2)
Ser[7]	7.52	3.79	3.74			
Gln[8]	7.70	4.04	2.05, 1.95	2.28		6.98, 6.60 (ε)
Lys[9]	7.45	3.94	1.64	1.46	2.20	3.17 (ε), 8.50 (ζ)
Arg[10]	8.05	4.03	1.83	1.72	3.11., 2.95	6.97
Lys[11]	7.79	4.07	1.94	1.54, 1.41	1.67	n.a.
Ile[12]	7.98	4.03	2.04	1.71, 1.29<br>1.03 (γCH_3)	0.94	
Thr[13]	7.97	4.33	4.40	1.46		
Leu[14]	7.98	4.42	1.84	1.67	0.97	
NH_2						7.24, 6.96
Decapeptide (D chain)						
Ac						2.05
Asp[1]	8.01	4.62	2.80			
Glu[2]	8.49	4.00	2.05	2.43		
Aib[3]	8.11		1.43			
Gln[4]	7.92	3.80	1.94, 1.85	2.28		7.27, 6.55 (ε)
Leu[5]	7.81	3.80	1.64, 1.56	1.30	0.80, 0.70	
Ser[6]	7.59	3.66	3.59, 3.48			
Aib[7]	7.42		1.20, 0.81			
Gln[8]	7.35	3.53	1.58, 1.50	2.03		6.98, 6.06 (ε)
Dab[9]	7.56	4.00	1,61	2.93, 2.81		7.55 (δ)
Arg[10]	7.576	4.00	1.83, 1.61	1.46, 1.27	3.04, 2.84	6.98
NH_2						7.16, 6.85

(n.a.) means not assigned

Table S2b: Co(III)-Lys9Dab-MC6*a-**1Δ** deuteroporphyrin ^{1}H assignments (ppm)

Deuteroporphyrin IX			
2 α,α'CH_2	4.98, 4.24	8H	9.55
18 α,α'CH_2	4.98, 4.44	10 H	10.63
2 β,β'CH_2	3.20, 3.10	12CH_3	3.84
18 β,β'CH_2	3.48, 3.01	13 H	9.36
3CH_3	3.71	15 H	10.37
5H	10.45	17CH_3	3.77
7 CH_3	3.83	20 H	10.64

Table S3a: Co(III)-Lys9Dab-MC6*a-**2Δ** ^{1}H chemical shift (ppm)

Tetradecapeptide (TD chain)						
Residue	NH	CαH	CβH	CγH	CδH	Others
Ac						2.00
Asp1	7.87	4.61	2.88			
Leu2	8.15	3.32	1.40, 0.53	1.16	0.91, 0.69	
Gln3	8.35	3.26	1.86, 1.77	2.12, 2.04		7.09, 6.49 (ε)
Gln4	7.77	3.68	1.92, 1.88	2.23		7.15, 6.52 (ε)
Leu5	7.59	3.56	0.95, 0.50	0.81	0.62, 0.16	
His6	6.30	2.32	1.49, 1.10			9.19 (δ1), -0.18 (ε1), -0.54 (δ2)
Ser7	7.47	3.83	3.76			
Gln8	7.60	4.12	2.12, 1.94	2.29		7.00, 6.58 (ε)
Lys9	7.34	3.98	1.69	1.53	1.46	3.14, 2.39 (ε) 8.30 (ζ)
Arg10	8.01	4.11	1.85	1.74, 1.55	3.14, 3.05	7.03
Lys11	7.81	4.12	1.93	1.54, 1.42	1.69	2.97 (ε), 7.60 (ζ)
Ile12	7.95	4.07	2.05	1.70, 1.31 1.05 (γCH_3)	0.96	
Thr13	7.94	4.36	4.40	1.05 (γCH3)45		
Leu14	7.95	4.42	1.84, 1.68	1.4585	0.97	
NH_2						7.26, 6.93
Decapeptide (D chain)						
Ac						2.07
Asp1	8.05	4.71	2.94, 2.90			
Glu2	8.39	4.05	2.11	2.49		
Aib3	8.08		1.47			
Gln4	7.95	3.83	1.96, 1.86	2.30		7.19, 6.55 (ε)
Leu5	7.80	3.87	1.66, 1.34	1.57	0.81, 0.72	
Ser6	7.59	3.62	3.56, 3.43			
Aib7	7.38		1.19, 0.58			
Gln8	7.22	3.46	1.64,1.50	2.01		6.97, 6.08 (ε)
Dab9	7.69	3.99	1.86, 1.62	3.05, 2.77		7.69 (δ)
Arg10	7.24	3.98	1.86, 1.63	1.44	3.06, 2.67	6.98
NH_2						7.24, 6.92

Table S3b: Co(III)-Lys9Dab-MC6*a-**2Δ** deuteroporphyrin ^{1}H assignments (ppm)

Deuteroporphyrin IX			
2 α,α' CH_2	4.98, 4.42	8H	9.47
18 α,α' CH_2	5.02, 4.23	10 H	10.61
2 β,β' CH_2	3.48, 3.03	12CH_3	3.87
18 β,β' CH_2	3.21, 3.14	13 H	9.41
3CH_3	3.79	15 H	10.29
5H	10.45	17CH_3	3.66
7 CH_3	3.77	20 H	10.65

The ^{1}H spectra (Figure S8) show a single set of resonances for the deuteroporphyrin protons, and two sets of resonances for the peptide chains. Resonances assignment was accomplished by 2D experiments (NOESY[11–13], TOCSY[14,15] and DQF-COSY[16]) by means of the sequential technique for both deuteroporphyrin and peptide protons.[17]

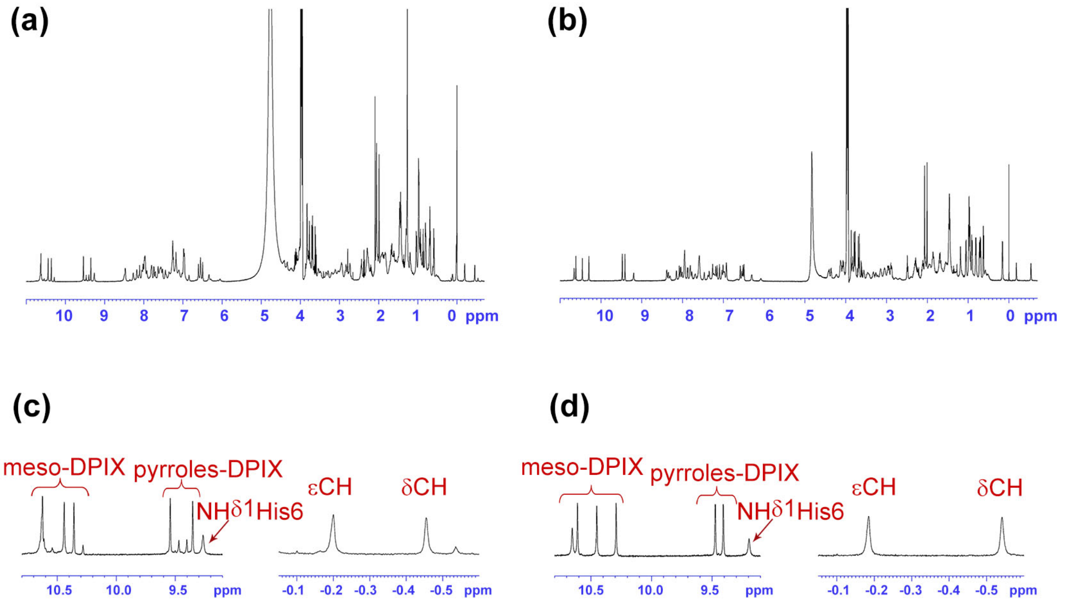

Figure S8. 1D ^{1}H NMR spectrum of Co(III)-Lys9Dab-MC6*a: (a) **1Δ** (2.0 10^{-4} M) and (b) **2Δ** (5.0 10^{-4} M) in H_2O/TFE solution (60/40, *v/v*) (pH = 4.6). (c) and (d) high field and low field regions of ^{1}H NMR spectrum of Co(III)-Lys9Dab-MC6*a-**1Δ** and 2**Δ**, respectively.

Histidine sidechain protons were unequivocally assigned. The δ-CH and ε-CH imidazole protons experience an extremely large ring current effect and appear in the high-field region of the spectrum (Fig S7c and S7d). The δ-CH resonances were easily identified on the basis of medium range NOE with the His6 ββ'-CH\ protons. In the TOCSY experiment the δ-CH signals for **1Δ** and **2Δ** at -0.45 ppm and -0.54 ppm, respectively, are correlated to ε-CH imidazole protons at -0.20 ppm and -0.18 ppm. These last resonances are in strong dipolar contact with resonances at 9.27 and 9.19 ppm, which was assigned to the imidazole δ-NHs of the histidine of the two tetradecapeptide chains.

All deuteroporphyrin proton resonances were unambiguously assigned, including those of the propionic groups that exhibited clearly distinct resonances. In particular, the deuteroporphyrin

assignment was easily performed following the NOE connectivities around the ring and starting from the 5H proton (at 10.45 ppm): it is the only proton in dipolar contact with the two methyl groups (3-CH_3 and 7-CH_3) (see Figure S1 for deuteroporphyrin numbering).

All the residues of the two peptide chains show αCH resonances significantly up-field shifted, relative to their random coil values (Figure S9). It is reasonable to assume that these deviations are due not only to the peptide secondary structures, but to deuteroporphyrin ring current too.

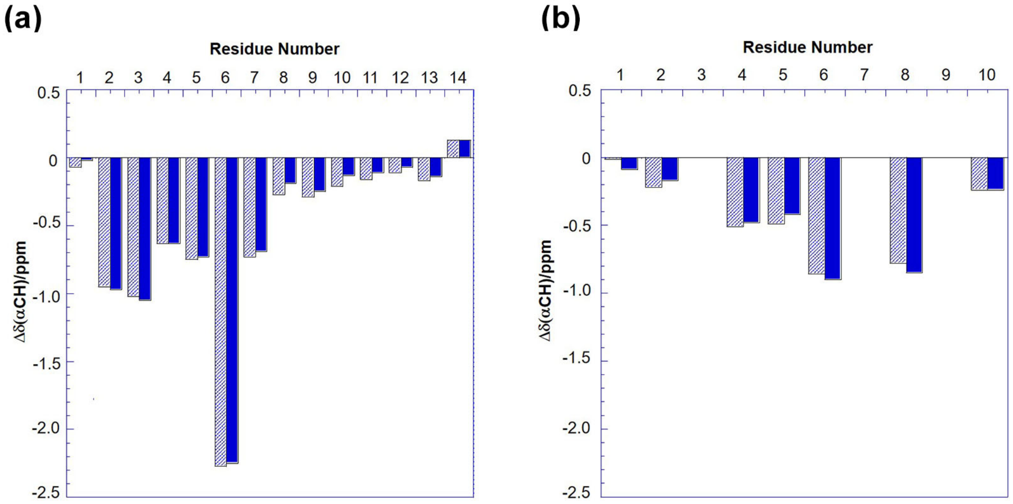

Figure S9. Chemical shift index Δδ(αCH) of (a) tetradecapeptide and (b) decapeptide chain of Co(III)-Lys9Dab-MC6*a-**1Δ** (dashed bars**)** and Co(III)-Lys9Dab-MC6*a-**2Δ** (solid bars).

A total of 513 and 589 NOESY cross peaks were assigned, integrated and transformed in upper distance limits with the program CALIBA[18] for **1Δ** and **2Δ**, respectively. Of these NOE upper distance limits, 155 and 143 for **1Δ** and **2Δ**, respectively were removed, because they do not effectively restrict the conformation. The final input for structure calculation consisted of 358 and 446 upper distance limit constraints for **1Δ** and **2Δ**, respectively.

To perform the simulated annealing, we included in the CYANA[19] standard library three non-standard residues.

As the CYANA library does not allow the introduction of metal ions, an artificial deuteroporphyrin IX residue, named DCO, was added in the residue library used by CYANA program. This residue consisted of a deuteroporphyrin IX ring containing a cobalt atom, defined as a dummy atom,

covalently bonded to the four pyrrole nitrogens and a CH_2 group bound at position 18 and 2 of the ring (see Figure S1 for deuteroporphyrin numbering).

In addition, to ensure the covalent linkage between the deuteroporphyrin moiety and both peptide chains, two non-standard residues, denoted by LYSD (inserted into the TD peptide sequence as LysD9) and DABD (inserted into the D peptide sequence as DabD9) were built and added to the CYANA library. These artificial residues contained, on the side chain, a carboxylic group involved in formation of amide bond through their nitrogen atoms (Nζ atom for LYSD and Nδ atom for DABD residue) and a methylene group. The carbon atom of the CH_2 group was linked to the carbon atom of the deuteroporphyrin methylene group at position 18 and 2. The link was defined by using appropriate upper and lower distance limits (d[LysD9 CP-DCO CAA] = 1.55 and 1.51 Å, d[DabD9 CP, DCO CAD] = 1.55 and 1.51 Å for the upper and lower limits, respectively).

The pentacoordinate geometry of the metal ion was accomplished by applying a "special covalent bond" with upper and lower distance limits of 2.1 and 1.8 Å between His6 Nε atom and the cobalt ion. No other constraints, except for the NOE constraints derived from NMR spectra, were imposed upon the side chain of the His6 axial ligand, to allow it to change the ring orientation with respect to the deuteroporphyrin plane axes. No hydrogen bond constraints were used in the structure calculations.

For both compounds, we were able to solve the structure of the cobalt complexes, obtaining an ensemble of 20 tightly clustered structures for each distereomers. The final family of each diastereomers converged to the same topology, with a root mean square deviation of 0.84 ± 0.30 Å and 0.99 ± 0.49 Å for the main chain atoms for compound **1Δ** and **2Δ**, respectively (Table S4).

Table S4. NMR and refinement statistics the final structures of Co(III)-Lys9Dab-MC6*a-**1Δ** and **2Δ**

	**1Δ**	**2Δ**
Parameter	Value	Value
Distance constraints		
Assigned NOE cross peaks	534	589
Nonredundant NOE upper distance limits	358	446
Intra-residue	123	162

Inter-residue	235	284
Sequential (|i – j| = 1)	91	91
Medium-range (|i – j| < 4)	74	106
Long-range (|i – j| > 5)	70	87
Structure statistics		
Violations (mean and s.d.)		
Distance constraints (Å)		
0.1 < d ≤ 0.2	12.15 ± 2.45	14.5 ± 2.3
0.2 < d ≤ 0.3	1.1 ± 0.4	2.2 ± 1.2
0.3 < d ≤ 0.4	0	0.2 ± 0.4
Max. distance constraint violation (Å)	0.22 ± 0.02	0.26 ± 0.033
Average pairwise r.m.s. deviation* (Å)		
All atoms	1.88 ± 0.46	2.22 ± 0.69
Backbone	0.84 ± 0.30	0.99 ± 0.49
Ramachandran statistics from PROCHECK-NMR‡		
Most favoured regions. %	86.4	90.9
Additional allowed regions. %	13.6	9.1
Generously allowed regions. %	-	-
Disallowed regions. %	-	-

*Average coordinates of the 20 energy-minimized conformers after the best fit superposition of the alpha carbons.

‡The program PROCHECK-NMR[20] was used to check the overall quality of the structures.

✓ *<u>Co(III)-Lys9Dab-MC6*a complex **1Δ** and **2Δ**</u> : diastereomer assignment*

The diastereomer assignment was validated by the analysis of the NOE contacts between the residues facing the DPIX (Leu2, Leu5, His6) of the TD chain and the DPIX ring. Leu5 residues, for both TD peptide chains, show more diagnostic contacts for diastereomer assignment. Figure S10 depicts qualitatively the final interpretation.

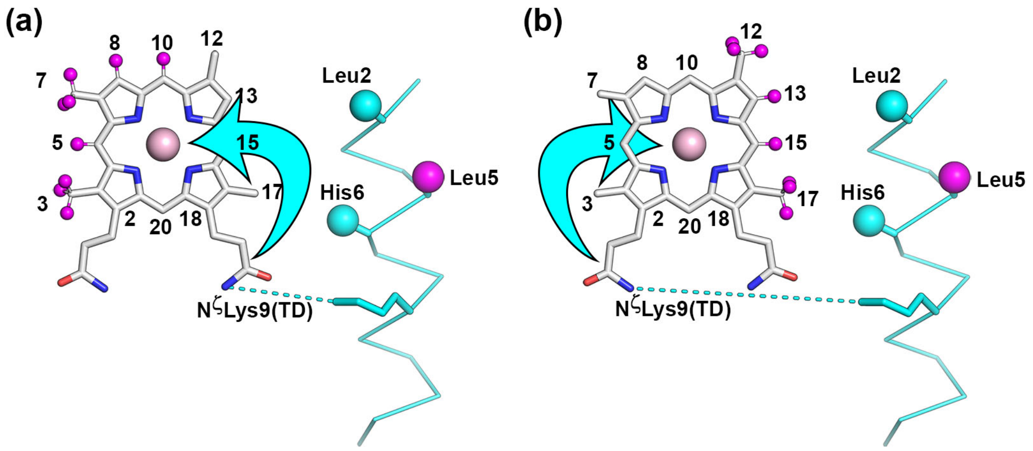

Figure S10. Assignment of the stereochemistry for **(a)** regioisomer **1**, and **(b)** regioisomer **2**. To fulfill NOE contacts between Leu5 and porphyrin, the helix formed by the TD chain must lie above and below the porphyrin plane for **1** and **2**, respectively, when the porphyrin is seen as depicted in this figure. Magenta spheres highlight porphyrin protons in contact with Leu5, whose Cβ atom is also highlighted in magenta. Cyan spheres highlight the Cβ atom of the other residues facing the porphyrin ring. Dashed line indicates the amide bond between Lys9 and propionyl groups either from position 18 or 2.

Section S4: Computational details for structural analysis of Co(III)-Lys9Dab-MC6*a-1Δ and 2Δ

✓ *Co(III)-Lys9Dab-MC6*a complex **1Δ** and **2Δ*** *: REM/RMD calculation*

RMD and REM protocols have been used as described in the main text. LEaP script has been used to type the newly parametrized DPIX, Aib, amidated Dab and Lys, as follows:

```
loadAmberPrep ../DAA/DAA_mod.prepin
loadamberparams ../DAA/frcmod2.DAA
loadamberparams ../DAA/frcmod1.DAA
loadAmberPrep ../LYA/LYA_mod.prepin
loadamberparams ../LYA/frcmod2.LYA
loadamberparams ../LYA/frcmod1.LYA
addAtomTypes{{NX N sp2}{NY N sp2}}
loadAmberPrep ../DCO/DCO_mod_2.prepin
loadAmberPrep ../AIB/AIB.prepin
loadAmberPrep ../OHX/OHX.prepin
loadamberparams ../OHX/frcmod.OHX
loadamberparams ../DCO/frcmod_allnew.DCO
loadamberparams ../DCO/frcmod_new.CO
loadAmberPrep ../DCO/CO.prepin
x = loadPDB  {%PATH}.pdb
bond x.21.NZ x.28.C5
bond x.9.ND x.28.CN
bond x.28.NA x.29.CO
bond x.28.NB x.29.CO
bond x.28.NC x.29.CO
bond x.28.ND x.29.CO
bond x.18.NE2 x.29.CO
bond x.30.O x.29.CO
saveAmberParm x {%PATH}.parm7 {%PATH}.rst7
quit
```

Leap command was then launched in the following general case:

```
tleap -f leaprc.ff99SB -f leap_{%modelN}.com #where modelN is a model-specific
                                               variable
```

All the parametrization files have been uploaded in a zenodo repository and are available for download (zenodo).

Table S5. Structural parameters calculated for each Co(III)-Lys9Dab-MC6*a-**1Δ** model.

	**Interhelical angle (°)**	**DPIX SASA ($Å^2$)**	**Co-D chain (CoM) distance (Å)**
**1Δ**_1	21.7	25.8	7.7
**1Δ**_2	27.0	24.2	7.9
**1Δ**_3	19.3	23.2	8.0
**1Δ** _4	29.6	23.6	8.0
**1Δ** _5	22.3	26.3	7.8
**1Δ** _6	14.2	22.2	7.7
**1Δ**_7	24.2	26.4	7.8
**1Δ**_8	21.4	23.9	7.8
**1Δ**_9	18.2	23.3	7.6
**1Δ**_10	27.9	25.4	7.9
**1Δ**_11	14.1	21.4	7.3
**1Δ** 12	19.9	25.6	7.9
**1Δ**_13	27.3	26.5	7.5
**1Δ**_14	5.8	25.0	8.0
**1Δ**_15	12.8	26.6	7.6
**1Δ**_16	23.0	25.6	7.8
**1Δ**_17	20.4	20.6	7.1
**1Δ**_18	28.4	25.5	7.2
**1Δ**_19	18.8	25.4	7.3
**1Δ**_20	23.0	23.7	7.6
**Average(SD)**	21(6)	24(2)	7.7(0.3)

Table S6. Structural parameters calculated for each Co(III)-Lys9Dab-MC6*a-**2Δ** model.

	**Interhelical angle (°)**	**DPIX SASA (Å²)**	**Co-D chain (CoM) distance (Å)**
**2Δ**_1	65.1	7.4	7.0
**2Δ**_2	66.9	7.9	7.2
**2Δ**_3	66.2	7.5	7.1
**2Δ**_4[a]	86.0	10.3	6.5
**2Δ**_5[a]	74.0	8.3	6.5
**2Δ**_6	52.0	4.9	7.7
**2Δ**_7[a]	78.9	10.0	6.6
**2Δ**_8[a]	98.4	8.9	6.5
**2Δ**_9	77.7	5.1	7.1
**2Δ**_10	60.1	14.7	8.3
**2Δ**_11	50.8	10.0	7.2
**2Δ**_12	90.0	3.1	6.6
**2Δ**_13	57.6	5.9	7.0
**2Δ**_14[a]	107.1	9.1	6.3
**2Δ**_15[a]	104.8	8.9	6.2
**2Δ**_16[a]	101.4	8.5	6.3
**2Δ**_17	64.9	6.0	7.5
**2Δ**_18	54.2	20.6	7.5
**2Δ**_19	63.4	6.0	7.5
**2Δ**_20[a]	113.9	9.4	6.4
**Average(SD)**	64(11)<br>96(13)	8(3)<br>9.2(0.7)	7.3(0.4)<br>6.4(0.1)

[a] this model belongs to family 2

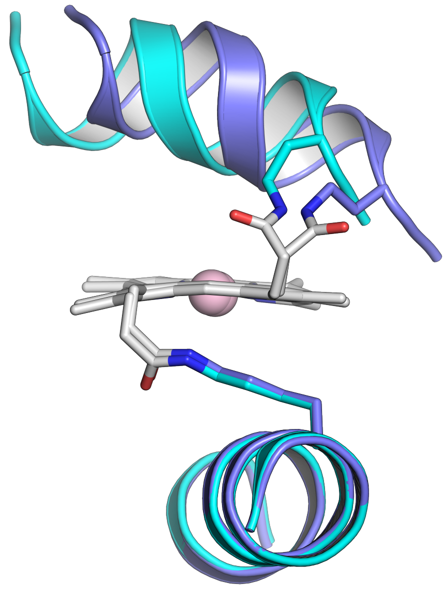

Figure S11. Representative models of the two structural families (model 1 and model 4 in cyan and blue cartoon, respectively) from **2Δ** bundle showing opposite orientation of the amide bond linking the Dab9 side chain to the propionyl 18 of the DPIX (grey stick).

Table S7. θ angle values. derived from NMR analysis, of Co(III)-Lys9Dab-MC6*a **1Δ** and **2Δ** compared to Fe(III) derivatives.

Compound	**1Δ** (°)	**2Δ** (°)
Co-Lys9Dab-MC6*a	-33	35
Fe-Lys9Dab-MC6*a	-19	45

Table S8. His6 second-shell interactions. Donor-acceptor distance and donor-acceptor-acceptor angle have been considered.

	**1Δ**		**2Δ**	
	**Nδ(His6)-O(Leu2) (Å)**	**Nδ(His6)-O(Leu2)-C(Leu2) (°)**	**Nδ(His6)-O(Leu2) (Å)**	**Nδ(His6)-O(Leu2)-C(Leu2) (°)**
1	2.71	134.6	2.70	118.0
2	2.70	132.7	2.72	119.1
3	2.71	134.3	2.72	120.0
4	2.71	134.2	2.72	119.3
5	2.71	134.8	2.69	118.1
6	2.71	134.0	2.71	119.9
7	2.72	135.1	2.71	121.6
8	2.70	133.1	2.72	121.0
9	2.71	134.1	2.74	117.4
10	2.70	134.2	2.73	121.7
11	2.69	133.0	2.69	119.5
12	2.71	132.5	2.72	118.1
13	2.71	134.6	2.71	118.3

14	2.69	132.0	2.72	120.5
15	2.72	133.1	2.72	119.6
16	2.73	137.4	2.70	121.8
17	2.70	137.7	2.72	118.2
18	2.70	132.4	2.73	115.9
19	2.71	133.5	2.73	120.7
20	2.69	135.6	2.72	117.2
**Average(SD)**	**2.70(0.01)**	**134.1(1.5)**	**2.72(0.01)**	**119.3(1.6)**

Section S5: References

(1) Maglio, O.; Chino, M.; Vicari, C.; Pavone, V.; Louro, R. O.; Lombardi, A. Histidine Orientation in Artificial Peroxidase Regioisomers as Determined by Paramagnetic NMR Shifts. Chem. Commun. 2021, 57 (8), 990–993. https://doi.org/10.1039/D0CC06676A.

(2) Vitale, R.; Lista, L.; Cerrone, C.; Caserta, G.; Chino, M.; Maglio, O.; Nastri, F.; Pavone, V.; Lombardi, A. An Artificial Heme-Enzyme with Enhanced Catalytic Activity: Evolution, Functional Screening and Structural Characterization. *Org. Biomol. Chem.* **2015**, *13* (17), 4859–4868. https://doi.org/10.1039/C5OB00257E.

(3) Nastri, F.; Lista, L.; Ringhieri, P.; Vitale, R.; Faiella, M.; Andreozzi, C.; Travascio, P.; Maglio, O.; Lombardi, A.; Pavone, V. A Heme–Peptide Metalloenzyme Mimetic with Natural Peroxidase-Like Activity. *Chem. Eur. J.* **2011**, *17* (16), 4444–4453. https://doi.org/10.1002/chem.201003485.

(4) Munro, O. Q.; Marques, H. M. Heme−Peptide Models for Hemoproteins. 1. Solution Chemistry of *N* -Acetylmicroperoxidase-8. *Inorg. Chem.* **1996**, *35* (13), 3752–3767. https://doi.org/10.1021/ic9502842.

(5) Yonetani, T.; Anni, H. Yeast Cytochrome c Peroxidase. Coordination and Spin States of Heme Prosthetic Group. *Journal of Biological Chemistry* **1987**, *262* (20), 9547–9554. https://doi.org/10.1016/S0021-9258(18)47968-9.

(6) Feis, A.; Marzocchi, M. P.; Paoli, M.; Smulevich, G. Spin State and Axial Ligand Bonding in the Hydroxide Complexes of Metmyoglobin, Methemoglobin, and Horseradish Peroxidase at Room and Low Temperatures. *Biochemistry* **1994**, *33* (15), 4577–4583. https://doi.org/10.1021/bi00181a019.

(7) *The Porphyrins: Volume 1, Structure and Synthesis, Part A*; Dolphin, D., Ed.; Porphyrins; Academic Press: New York, 1978.

(8) D'Auria, G.; Maglio, O.; Nastri, F.; Lombardi, A.; Mazzeo, M.; Morelli, G.; Paolillo, L.; Pedone, C.; Pavone, V. Hemoprotein Models Based on a Covalent Helix–Heme–Helix Sandwich: 2. Structural Characterization of Co^{III} Mimochrome I Δ and Λ Isomers. *Chemistry A European J* **1997**, *3* (3), 350–362. https://doi.org/10.1002/chem.19970030306.

(9) Firpo, V.; Le, J. M.; Pavone, V.; Lombardi, A.; Bren, K. L. Hydrogen Evolution from Water Catalyzed by Cobalt-Mimochrome VI*a, a Synthetic Mini-Protein. *Chem. Sci.* **2018**, *9* (45), 8582–8589. https://doi.org/10.1039/C8SC01948G.

(10) Wishart, D. S.; Sykes, B. D.; Richards, F. M. Relationship between Nuclear Magnetic Resonance Chemical Shift and Protein Secondary Structure. *Journal of Molecular Biology* **1991**, *222* (2), 311–333. https://doi.org/10.1016/0022-2836(91)90214-Q.

(11) Jeener, J.; Meier, B. H.; Bachmann, P.; Ernst, R. R. Investigation of Exchange Processes by Two-dimensional NMR Spectroscopy. *The Journal of Chemical Physics* **1979**, *71* (11), 4546–4553. https://doi.org/10.1063/1.438208.

(12) Kumar, A.; Ernst, R. R.; Wüthrich, K. A Two-Dimensional Nuclear Overhauser Enhancement (2D NOE) Experiment for the Elucidation of Complete Proton-Proton Cross-Relaxation Networks in Biological Macromolecules. *Biochemical and Biophysical Research Communications* **1980**, *95* (1), 1–6. https://doi.org/10.1016/0006-291X(80)90695-6.

(13) Kumar, A.; Wagner, G.; Ernst, R. R.; Wüthrich, K. Buildup Rates of the Nuclear Overhauser Effect Measured by Two-Dimensional Proton Magnetic Resonance Spectroscopy: Implications for Studies of Protein Conformation. *J. Am. Chem. Soc.* **1981**, *103* (13), 3654–3658. https://doi.org/10.1021/ja00403a008.

(14) Bax, A.; Davis, D. G. MLEV-17-Based Two-Dimensional Homonuclear Magnetization Transfer Spectroscopy. *Journal of Magnetic Resonance (1969)* **1985**, *65* (2), 355–360. https://doi.org/10.1016/0022-2364(85)90018-6.

(15) Griesinger, C.; Otting, G.; Wüthrich, K.; Ernst, R. R. Clean TOCSY for Proton Spin System Identification in Macromolecules. *J. Am. Chem. Soc.* **1988**, *110* (23), 7870–7872. https://doi.org/10.1021/ja00231a044.

(16) Piantini, U.; Sorensen, O. W.; Ernst, R. R. Multiple Quantum Filters for Elucidating NMR Coupling Networks. *J. Am. Chem. Soc.* **1982**, *104* (24), 6800–6801. https://doi.org/10.1021/ja00388a062.

(17) Wüthrich, K. *NMR of Proteins and Nucleic Acids*; The George Fisher Baker non-resident lectureship in chemistry at Cornell University; Wiley: New York, 1986.

(18) Güntert, P.; Braun, W.; Wüthrich, K. Efficient Computation of Three-Dimensional Protein Structures in Solution from Nuclear Magnetic Resonance Data Using the Program DIANA and the Supporting Programs CALIBA, HABAS and GLOMSA. *Journal of Molecular Biology* **1991**, *217* (3), 517–530. https://doi.org/10.1016/0022-2836(91)90754-T.

(19) Güntert, P. Automated NMR Structure Calculation With CYANA. In *Protein NMR Techniques*; Humana Press: New Jersey, 2004; Vol. 278, pp 353–378. https://doi.org/10.1385/1-59259-809-9:353.

(20) Laskowski, Roman, A.; Rullmann, J. Antoon C.; MacArthur, Malcolm W.; Kaptein, R.; Thornton, Janet M. AQUA and PROCHECK-NMR: Programs for Checking the Quality of Protein Structures Solved by NMR. *J Biomol NMR* **1996**, *8* (4). https://doi.org/10.1007/BF00228148.
